# Common Cause Versus Dynamic Mutualism: An Empirical Comparison of Two Theories of Psychopathology in Two Large Longitudinal Cohorts

**DOI:** 10.1177/21677026231162814

**Published:** 2023-05-25

**Authors:** Michael E. Aristodemou, Rogier A. Kievit, Aja L. Murray, Manuel Eisner, Denis Ribeaud, Eiko I. Fried

**Affiliations:** 1 Department of Clinical Psychology, Leiden University; 2 Donders Center for Medical Neurosciences, Radboud University Medical Center; 3 MRC Cognition and Brain Sciences Unit, University of Cambridge; 4 School of Philosophy, Psychology and Language Sciences, University of Edinburgh; 5 Institute of Criminology, University of Cambridge; 6 Jacobs Center for Productive Youth Development, University of Zurich

**Keywords:** mutualism, common cause, p factor, depression, longitudinal modeling, comorbidity, ontology, theory

## Abstract

Mental disorders are among the leading causes of global disease burden. To respond effectively, a strong understanding of the structure of psychopathology is critical. We empirically compared two competing frameworks, dynamic-mutualism theory and common-cause theory, that vie to explain the development of psychopathology. We formalized these theories in statistical models and applied them to explain change in the general factor of psychopathology (p factor) from early to late adolescence (*N* = 1,482) and major depression in middle adulthood and old age (*N* = 6,443). Change in the p factor was better explained by mutualism according to model-fit indices. However, a core prediction of mutualism was not supported (i.e., predominantly positive causal interactions among distinct domains). The evidence for change in depression was more ambiguous. Our results support a multicausal approach to understanding psychopathology and showcase the value of translating theories into testable statistical models for understanding developmental processes in clinical sciences.

Theories about the genesis and development of psychopathology percolate into all aspects of clinical research and practice. The common-cause theory, which posits that a singular causal mechanism underpins the broad majority of psychiatric syndromes, has driven the field in past and recent decades (van Praag, 2000; Watts et al., 2019; Zachar, 2000). Originally, the common-cause theory operated *within* traditional syndromes, such as major depression, and fueled a long tradition of attempts to isolate the hidden causes and treat them. Following criticisms targeted at the high degree of overlap in the presentation of theoretically distinct syndromes (Hyman, 2021; Kotov et al., 2017; Lahey et al., 2017; McGorry et al., 2018), attempts to restructure psychopathology started to gain traction, and the common-cause theory found a new application: to explain the observation that one *statistical* dimension summarizes people’s proclivity to exhibit all major forms of psychopathology. This dimension is termed the “p factor” of psychopathology and has different interpretations (Fried, 2020; Sprooten et al., 2022). The common-cause interpretation, perhaps best introduced by Caspi and Moffitt (2018, p. 3), interprets the p factor as the consequence of a unitary cause that underlies any and all symptoms of mental illness (Watts et al., 2019, p. 2).

An alternative interpretation of the statistical p factor, such as shared variance among mental-health symptoms, comes from the dynamic-mutualism theory of psychopathology (hereafter referred to as mutualism theory), which is nested in the broader literature on the network theory of mental disorders. The mutualism theory postulates that syndromes form through the causal interactions among their constituent symptoms across development (Borsboom, 2017; Borsboom & Cramer, 2013; McNally, 2016; van der Maas et al., 2006). Therefore, the shared variance captured by the statistical p factor does not necessarily reflect a shared cause. It is plausible that the p factor is the product of causal interactions among disparate causes, which give rise to the same covariation structure that is aptly summarized by a single statistical dimension (van Bork et al., 2017). That is, the same outcome can arise from different mechanisms that support different methods for the way mental illness is measured, studied, and treated. A causal p factor would imply that rapid progress entails treating all psychopathology as a single disease, with the potential for a common intervention for all patients (Caspi & Moffitt, 2018). Mutualism, on the other hand, proposes that the way forward is through a granular understanding of causally active symptoms and their interactions, which form the statistical p factor. Mutualism predicts that a common treatment for all patients would be more limited in its effectiveness because heterogeneity in symptoms/syndromes entails heterogeneity in causes. Instead, interventions designed to identify and address the temporal unfolding of causal interactions between symptoms/syndromes hold greater promise under mutualism. This distinct treatment implication has been proposed as a test of the two theoretical frameworks (Fried & Cramer, 2017). That is, intervening on a symptom will either affect symptoms that are causally connected to the perturbed symptom, in line with mutualism, or will have no effect on other symptoms because their common cause is unaffected, in line with the common-cause theory.

The debate between the mutualism and common-cause theories also remains unresolved at the level of traditional syndromes, such as major depression. The bulk of past treatment and research efforts have been designed to address depression as originating from a single causal mechanism, such as imbalances in the brain’s serotonin system (Coppen, 1967; Cowen, 2008). Numerous neurobiological alterations have been proposed as the driving force behind major depression (Belmaker, 2004; Etkin et al., 2015; Hyman & Nestler, 1996; Kupfer et al., 2012; Schildkraut, 1965), and many treatments were designed to target its hypothesized core (Otte et al., 2016). However, “no established mechanism can explain all aspects of the disease” (Otte et al., 2016, p. 1), and the effectiveness of treatments targeting hypothesized common causes of depression is currently unclear or unsatisfactory (Bobo et al., 2016; Lv et al., 2019; Munkholm et al., 2019). Under mutualism theory, the inability to identify a singular cause is expected because depression emerges from the interactions among its constituent parts. This suggests that the goal of targeting a common cause to treat major depression is not tenable because there is no common cause independent of symptoms (Borsboom et al., 2019). Moreover, if symptoms are causally active, then symptom heterogeneity becomes substantively meaningful (Fried, 2015). This means that the heterogeneous presentations of depression would require a modified treatment approach.

Across the diverging breadths of symptoms covered by general psychopathology and major depression, the degree to which theoretical mechanisms contribute to the genesis and sustenance of psychopathological phenotypes matters greatly. The evidence to test the theories is, however, challenging to acquire and interpret. Two main obstacles complicate endeavors to ascertain the degree to which the common-cause theory and the mutualism theory are involved in the development of both transdiagnostic psychopathology and single disorders. First, the statistical proxies of the common-cause theory and the mutualism theory (i.e., common-factor models and network models) are difficult to distinguish in typical studies because they offer equivalent descriptions of cross-sectional data (Fried, 2020; Kruis & Maris, 2016; Marsman et al., 2015; but see van Bork et al., 2021). Second, both theories offer verbal and imprecise descriptions of their proposed mechanisms (Fried, 2020; Robinaugh et al., 2019, 2021). The inherent imprecision of their verbal nature makes it hard to conclude whether observations are concordant with theory because it is not clear what a theory entails to begin with (Fried, 2020; Fried et al., 2021). A developmental perspective holds the potential to remedy these issues. Each theory makes distinct, if not perfectly precise, predictions about dynamic behavior that researchers can exploit using longitudinal data and quantitative models to make progress toward a more nuanced understanding of the verisimilitude of each theory.

Only a few studies have compared the two theories using a combination of statistical models and longitudinal data, and none found clear evidence for either theory (Greene & Eaton, 2017; McElroy et al., 2018; Murray et al., 2016; Snyder et al., 2017). Given the complexity of the topic, several challenges point to the merit of further investigation. First, some prior studies relied on few assessments that covered a modest time span (e.g., 18–24 months, two waves; Greene & Eaton, 2017; Snyder et al., 2017), limiting the amount of symptom change and the applicability of their results to a narrow time span. Second, other studies sampled from a wide age range that covers divergent life periods (e.g., Greene & Eaton, 2017), which may be associated with different developmental mechanisms (Kievit, 2020; Kievit et al., 2017). Third, studies used cross-lagged panel models (e.g., McElroy et al., 2018), which may fail to represent within-persons relationships over time if stable individual differences are present (Hamaker et al., 2015). Moreover, this sample relied on maternal reports that are not necessarily aligned with children’s responses once they can self-report (Waters et al., 2003). Fourth, most studies did not assess longitudinal invariance (Greene & Eaton, 2017; McElroy et al., 2018; Murray et al., 2016), which leaves open the question of whether the p factor or other statistical dimensions modeled in the studies qualitatively shift across time. Finally, and most important, no prior studies formalized and directly compared the two developmental mechanisms in a longitudinal context.

We aim to supplement past efforts by directly comparing the developmental mechanisms posited by the common-cause theory and the mutualism theory. We do so by translating these two theories into statistical models that impose theory-consistent assumptions on the data. More specifically, we translate fundamental predictions made by each theory into latent-change-score (LCS) models (Kievit et al., 2018; McArdle et al., 2002; McArdle & Hamagami, 2001) that are well suited to study temporal dynamics (Ferrer & McArdle, 2010). Two properties of LCS models allow us to operationalize the mechanism through which dynamic-mutualism theory proposes the positive manifold of psychopathology manifests. First, we can test a core assumption of dynamic mutualism that more psychopathology in a given domain will lead to more change in psychopathology in a distinct domain. This is done using the proportionality parameters in LCS models, which translate this assumption into a testable prediction: Specifically, people with higher scores on psychopathology at a given time point will show greater change in psychopathology at the subsequent time point, controlling for the association between psychopathology and change in the same domain. Second, LCS models allow us to specify accumulating short-term dynamics and are thus well aligned with a dynamical-systems approach (Càncer et al., 2021; Usami et al., 2019), which subsumes dynamic mutualism (van der Maas et al., 2006). That is, all changes accumulate and affect later occasions (McArdle, 2009). We further extend prior work by comparing the ability of the two theoretical accounts to explain change across two domains of psychopathology (p factor and major depressive disorder [MDD]) at two distinct developmental periods using two distinct longitudinal data sets (Zurich Project on the Social Development of Children and Youths [z-proso]: *n* = 1,428; Survey of Health, Ageing and Retirement in Europe [SHARE]: *n* = 6,443). We have two main research questions, each corresponding to a different data set:

*Research Question 1*: Which data-generating mechanism produces expected responses that better align with the observed pattern of responses in symptoms commonly summarized by the p factor from early to late adolescence—common-cause theory or mutualism theory?
*Research Question 2*: Which data-generating mechanism produces expected responses that better align with the observed pattern of responses in symptoms commonly summarized by major depression—common-cause theory or mutualism theory?

To increase the accessibility of our proposed method for theory evaluation, we refer readers interested in progressively gaining a deeper understanding of LCS models and competing and complementary analytical frameworks to the rich literature of tutorials covering diverse didactic methods and reproducible code in openly available software (Kievit et al., 2018; McArdle, 2009; McCormick et al., 2021; Usami et al., 2019; Zyphur et al., 2020).

## Method

### Transparency and openness

This study involved analyses of existing data rather than new data collection. We report all data-preprocessing steps, including exclusions and transformations. We report our sample characteristics and detail the analyzed measurement scales. Code for all our analyses, preprocessing steps, and data visualization are available at https://osf.io/a4ywe/?view_only=498f5640c18847bea3ac6a9b0b596821. Data were analyzed using R (Version 4.1.3; R Core Team, 2013). We include a supplement that thoroughly documents our results. We used two data sets that include sensitive information about human subjects. The SHARE data are open access. Interested researchers can access the data through http://www.share-project.org/data-access.html. The z-proso data are largely open access and will be made available on SWISSubase in 2023. Interested researchers are invited to contact the study directors to join the network and to access the data and documentation. All our confirmatory analyses are preregistered. Exploratory analyses are stated as such throughout the article. Deviations from our preregistration and corresponding rationale can be found in Table S8 in the Supplemental Material available online.

### Data Set 1: z-proso

#### Participants

The sample was obtained from the z-proso. The z-proso is a longitudinal cohort and intervention study that focuses on the development of adaptive and maladaptive social behaviors. Data are treated as observational because early interventions had no substantial effects on children (e.g., Averdijk et al., 2016; Malti et al., 2011). The study population consists of three quarters of all children that started primary school in the academic year of 2004–2005 in Zurich. This consists of 1,675 children from 56 public primary schools. Approximately half of the sample identified as male, and the other half identified as female (52% male, 48% female). The sample is ethnically diverse; the Swiss majority constitutes 37% of the sample, and 63% comes from 87 different countries. At age 15, near the end of compulsory education, 20% went to grammar school, 41% went to secondary school A (upper tier), 37% went to secondary school B or C (lower tier), and 2% attended special education. We assessed data from the four most recent measurement waves collected to date. This includes data from 1,482 children, 88% of the original target sample. Missing data (≈11%) was dealt with using full information maximum likelihood (FIML; N. L. Eisner et al., 2019; Enders & Bandalos, 2001). Children’s median age at each wave is approximately 13, 15, 17, and 20 years. For more detailed information regarding data collection and sample characteristics, see M. Eisner et al. (2012) and Ribeaud et al. (2022).

#### Measures

Psychopathology symptoms were measured using an adaption of the self-report version of the Social Behavior Questionnaire (SBQ; Tremblay et al., 1991). The z-proso version adds several items to enhance the measurement of psychopathology and improve developmental appropriateness as children move through different life periods. The original 3-point scale was converted to a 5-point Likert scale (*never* to *very often*), and the questionnaire was administered in German. Prior psychometric analyses have “generally supported the factorial validity, criterion validity, and reliability of the SBQ items” (Murray et al., 2019, p. 1236). We examined 42 items that were consistently measured over four waves. The analyzed items assess the constructs of prosociality, aggression, oppositionality, depression, anxiety, and attention-deficit/ hyperactivity disorder (ADHD). All measured domains refer to the frequency of behavior in the past year, except for anxiety and depression items, which refer to frequency in the last month. All items were treated as continuous. Continuous methodology performs as well as categorical methodology when a 5-point scale is used and response distributions are approximately symmetrical (Rhemtulla et al., 2012). Prosociality items were recoded so that higher scores indicate lower levels of prosociality.

#### Statistical analyses (Data Set 1: z-proso)

The following segment describes the specification, estimation, and assessment of the structural equation models used to compare the two theories. First, we describe the specification of the measurement models and the structural models. Thereafter, we report our choice of estimator and the fit indices used to compare models. Finally, we describe how we tested for measurement invariance.

##### Exploratory factor analysis

We used an exploratory process to estimate the measurement models. First, we selected four first-order factors (internalizing, externalizing/aggression, prosociality, ADHD) based on previous work (Murray et al., 2016, 2019). Second, we used exploratory factor analysis (EFA) on each wave to check whether a four-factor solution had replicable item content. We decided to use EFA to check the stability of item content because the work by Murray et al. (2016, 2019), from which we drew our four factors, sampled from a partially different age range (5–15 vs. 11–17) and omitted some SBQ items from their analyses. Prior studies have shown that differences in age and item content may affect the stability of item content. For instance, Wittchen et al. (2009) showed that a popular three-factor structure was not robust to the addition of symptom items representing a wider breadth of psychopathology and was not stable across development. EFA was conducted using the *psych* package in R (Revelle, 2019), and factors were extracted using minimal residual extraction with oblique rotation. The content of the four factors was almost identical across the first two waves but differed greatly compared with the last two waves (see Tables S11–S14 in the Supplemental Material). We decided to use the factors from the first two waves because they closely matched the factors from previous studies using the SBQ (Murray et al., 2016).

##### Confirmatory factor analysis

**Fig. 1.**
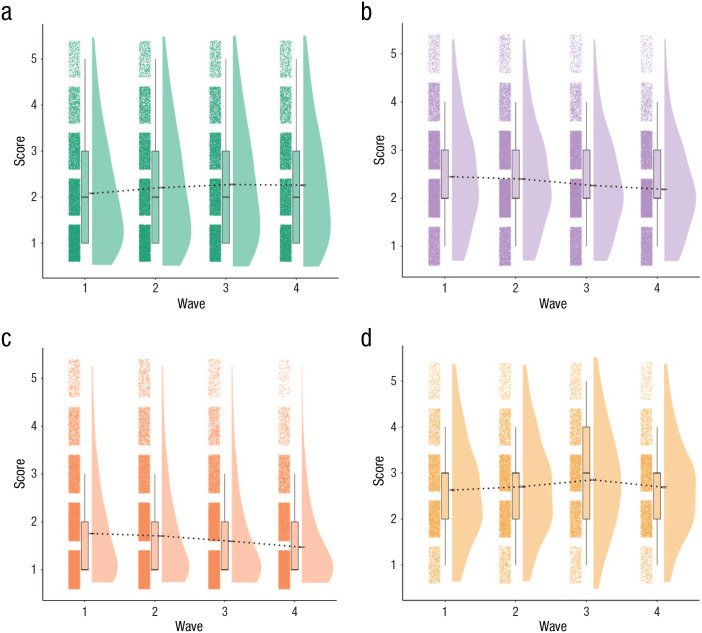
Rain-cloud plots of items grouped by factor structure of Zurich Project on the Social Development of Children and Youths (z-proso) data set. Items are rated on a 5-point Likert scale. Higher scores on all domains indicate a higher degree of psychopathology. The black horizontal bars at the base (approximately middle) of each density plot represent the standard error of the mean. The dashed black lines passing through subsequent waves indicate changes in mean severity of psychopathology over time.

All analyses were conducted using the *lavaan* package in R (Rosseel, 2012). The four specific factors obtained through EFA were used to specify the confirmatory factor models. For the common-cause model, confirmatory factor models were specified for each wave. These models incorporate a five-factor structure composed of four, mutually correlated, first-order factors (internalizing, externalizing/aggression, prosociality, ADHD) and a second-order factor (p factor; for details on the choice of a higher-order factor model to estimate the p factor, see Methodological Details in the Supplemental Material). The second-order factor summarizes the variance shared by the first-order factors. For the dynamic-mutualism model, confirmatory factor models were specified for each wave. These are composed of four correlated first-order factors (internalizing, externalizing/aggression, prosociality, ADHD; Fig. 1).

##### Structural models

To compare competing theoretical mechanisms, we specified different LCS models (Kievit et al., 2018; McArdle et al., 2000; McArdle & Hamagami, 2001). The key notion in LCS models is that successive differences between measures can be used to calculate change scores. If there is a basic autoregressive model in which the scores of person *i* for construct *y* at time *t* are a function of the person’s score at the previous time point (β*y_t_*_-1,*i*_) and some residual ζ,

(1) $$y_{t,i}=\;\beta y_{t-1,i}+\;\zeta_{t,i}.$$

then setting the regression slope (β) to equal 1 (Equation 2) allows us to conceptualize the residual as the difference between *y_t_*_,*i*_ and *y_t_*_-1,*i*_ (Equation 3), representing the change score Δ*y_t,i_* (Equation 4). The algebraic process is depicted below. In Equation 2, we set the linear effect of *y_t_*_-1,*i*_ on *y_t,i_* to 1. If we move *y_t-_*_1__,*i*_ to the left side, we get Equation 3. Equation 3 is identical to Equation 4; we simply relabeled the residual to clarify that it represents the change in a person’s scores between two successive time points, that is, ζ_*t,i*_ = Δ*y_t,i_* = *y_t,i_ – y_t-_*_1,*i*_.

(2) $$y_{t,i}=y_{t-1,i}+\;\zeta_{t,i}$$

(3) $$\zeta_{t,i}=y_{t,i}-y_{t-1,i}$$

(4) $$\Delta y_{t,i}=y_{t,i}-y_{t-1,i}.$$

We then defined a LCS factor Δη_*t,i*_, with a factor loading equal to 1. The residual variance of the corresponding observed score was set to 0 because all of the residual variance is captured by the LCS. This step allows us to extract more information about the change process than would be available using the observed change score. First, we can assess the average amount of change that transpired during a specific time interval (e.g., between Time 1 [T1] and Time 2[T2]) in our population. Second, we can assess how much individuals differ from each other in the amount of change they manifest by adding a variance component to the latent change score. Third, we can assess the extent to which the degree of change at T2, for example, is proportional to the baseline level, in this example, T1, of a person’s scores on the same attribute using a self-feedback parameter (φ). Note this captures the association between individual differences in change and individual differences in prior scores. This is different from the association between scores at two successive time points, which is conventionally set to 1 in LCS models.

Next, we extended the univariate LCS model to a multivariate LCS model. This allows us to do two crucial things. First, we can model change scores in multiple domains. Second, we can add an additional regression parameter that captures the degree to which change in a given domain at T2, for example, is associated with the baseline level of another domain, in this case, the score at T1 (McArdle et al., 2002). This regression parameter is called a coupling parameter (γ) and captures reciprocal relations between two distinct domains. Hence, change scores in a multivariate LCS model are modeled as a function of two parameters (Equation 5): a self-feedback process (ϕ) that captures the extent to which change in a given domain (e.g., Δ*y*1) depends on the prior state in that domain (ϕ1*y*1_*t*-1_), with ϕ1 denoting the effect size of self-feedback during the specified time interval, and a coupling parameter (γ), which captures the extent to which change in one domain Δ*y*1 at time *t* depends on the score of another domain *y*_2_ at the preceding time point *t* − 1, with γ1 denoting the effect size of coupling during that particular time interval (i.e., γ1 × y2_*t*-1,*i*_).

(5) $$\Delta \eta 1_{t,i}=\varphi {1y1}_{t-1,i}+\;\gamma 1y2_{t-1,i}.$$

##### Common-cause model

**Fig. 2.**
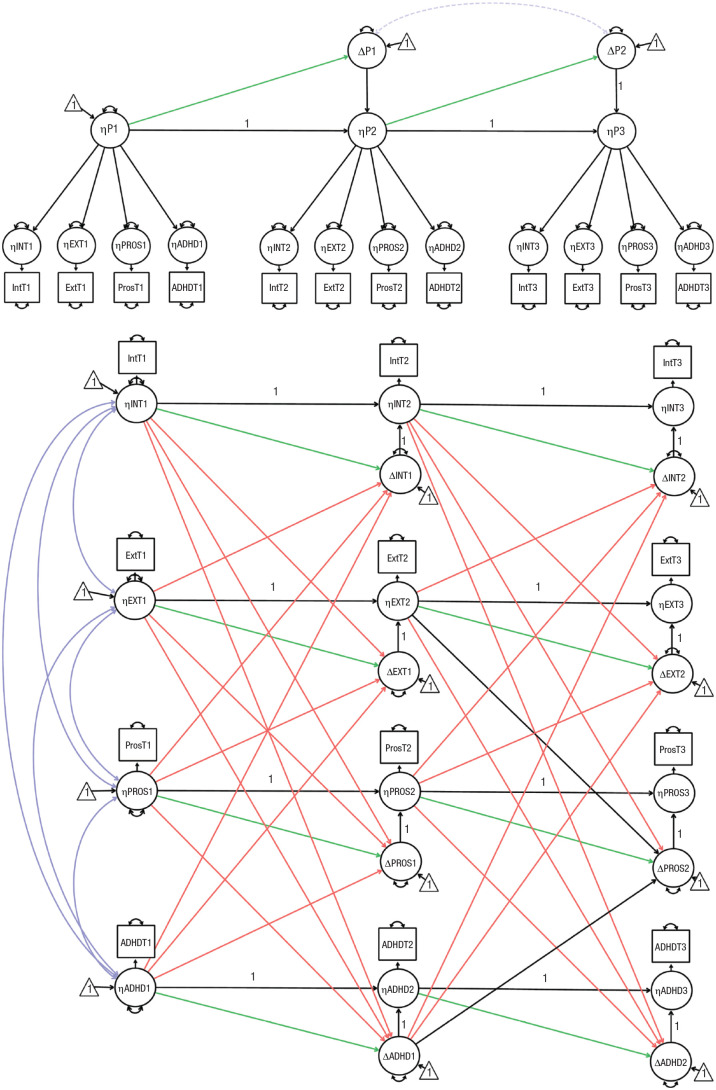
Illustration of common-cause model (top) and dynamic mutualism model (bottom) for Zurich Project on the Social Development of Children and Youths data. Circles indicate latent variables, rectangles indicate observed variables, and triangles indicate intercepts. Double-headed arrows indicate covariances (purple) and variances (black). Dashed lines show the parameters that were included only in the exploratory analyses. Single-headed arrows denote regressions. Green and orange single-headed arrows are the focal parameters for our model comparison. Green single-headed arrows indicate self-feedback parameters (β). Orange single-headed arrows indicate coupling parameters (γ). A “1” shows that the parameter has been constrained to 1. The illustration depicts only a limited number of waves and one observed variable per factor for visual clarity.

The p factor is the mechanism that drives development over the assessed time span. Thus, each person’s developmental trajectory is created through the accumulation of latent changes in general psychopathology (p factor) over time. Scores on lower-order latent dimensions of psychopathology, such as the internalizing dimension, are caused by changes in the p factor. Changes in lower-order dimensions, in turn, cause changes in symptoms of psychopathology. This process is modeled using a higher-order factor model, which reflects the mediated (indirect) effect of the p factor on observed psychopathology. In sum, the mechanism of change operates at the higher latent level of the p factor, and its effects trickle down to our observations. A univariate LCS model is used to specify the change process at the level of the p factor. Change in the p factor at each time point is influenced by two factors. First is a self-feedback parameter (ϕ, green arrow in Fig. 2), which relates the rate of change in the p factor at time *t* to the level of the p factor at the previous time point. A positive self-feedback parameter reflects accelerated growth, whereas negative self-feedback is indicative of dampening our statistical artifacts, such as regression to the mean. Second is the indirect effect of prior changes that flow through the fixed-unit paths. This causal flow can be observed by following the fixed-unit paths in Figure 2 (e.g., pfactor_*t*2_ = 1 × Δpfactor_*t*2_ → *p*factor_*t*3_ = 1 × *p*factor_*t*2_; McArdle, 2009). The hypothesis that the “*p* factor is a stable generalized liability to develop any and all forms of psychopathology across the life course” (Caspi & Moffitt, 2018, p. 840) would be consistent with, on average, nonsignificant self-feedback parameters. That is, the average (change score) residual variance after accounting for autoregression between two successive time points should be null. Alternatively, we can take an agnostic stance on the stability of the p factor but still hypothesize that it is a common cause for all types of psychopathologies. This can (to an extent) be tested through the residual covariance between change scores across time points. These residual covariances reflect a mismatch between the tempo of measurement and the temporal unfolding of the mechanism and/or the effect of unmeasured factors that influence development (Curran et al., 2014; Hofman et al., 2018). Null residual covariances between changes scores would be in line with a common cause interpretation of the p factor. We estimated the mean and variance of the LCSs at each wave. Self-feedback parameters were freely estimated across waves, and residual change score covariances were set to zero to test both possibilities. The mean and variance of the p factor were estimated at T1, and equality was constrained over time so that any actual change is reflected as change scores. We allowed residual terms to covary between time points for each observed variable with itself to allow indicator-specific variance (Kievit et al., 2017). We imposed measurement invariance over time. The common-cause model predicts the p factor will be invariant over time, reflecting a lack of qualitative changes in the mechanism causing psychopathology at each time point.

##### Dynamic-mutualism model

The development of psychopathology is driven by changes in four latent dimensions: internalizing, externalizing, prosociality, and ADHD. Different subsets of manifest psychopathology are directly caused by their respective superordinate latent dimension (e.g., ADHD symptoms are caused by ADHD). Symptoms across distinct dimensions cause each other, indirectly, through the causal interactions that happen among their superordinate latent dimensions (e.g., ADHD causes change in internalizing, which directly causes change in its symptoms). We modeled this structure using a confirmatory-factor model in which four first-order factors (representing the latent dimensions) had direct associations with a unique subset of symptoms (Fig. 2). The mechanism of change happens at the latent level, and its effects trickle down to our observations. We modeled the change process using a multivariate LCS model. Change in any latent dimension, at each time point, is determined by three factors. First is a self-feedback parameter (ϕ, green arrow in Fig. 2), which relates the rate of change in a given dimension (e.g., ADHD) at time *t* to the level of the same dimension (ADHD again) at the previous time point. A positive self-feedback parameter reflects accelerated growth, whereas negative self-feedback is indicative of dampening or statistical artifacts, such as regression to the mean. Second is coupling parameters (γ), which reflect the influence of severity in a given dimension (e.g., ADHD) at the preceding time *t* − 1 on the rate of change in other dimensions at time *t*. The coupling parameters reflect the core mechanism of dynamic mutualism (i.e., causal interactions between distinct dimensions of psychopathology) and are related to the M matrix in the mutualism model (Hofman et al., 2018; van der Maas et al., 2006). Third is the indirect effect of prior changes that flow through the fixed-unit paths. This causal flow can be observed by following the fixed-unit paths in Figure 2 (e.g., ADHD_*t*2_ = 1 × ΔADHD_*t*2_ → ADHD_*t*3_ = 1 × ADHD_*t*2_; McArdle, 2009). The mutualism model predicts predominantly positive bidirectionality between dimensions (i.e., most coupling parameters should be positive). If the tempo of sampling does not match the temporal unfolding of the developmental mechanism but positive causal interactions are still the dominant driving force of development in between the assessments, the coupling parameters should be positive, but the effect sizes will be inflated. We estimated the mean and variance of the LCSs at each wave. The mean and variance of the four first-order factors were estimated at T1, and equality was constrained over time so that any actual change is reflected as change scores. Both self-feedback parameters and coupling parameters were freely estimated across time points to allow the association between the current state and subsequent change to quantitively differ across development. All four latent dimensions were allowed to correlate at T1. To allow indicator-specific variance, we allowed residual terms to covary between time points for each observed variable with itself (Kievit et al., 2017). Dynamic-mutualism theory allows for unmodeled symptoms/disorders to affect change in psychopathology; therefore, latent change factors were allowed to correlate within and between time points. We imposed measurement invariance over time.

##### Model fit and comparison

FIML with robust standard errors was used to deal with missingness and nonnormality. We relied on the following indices for the assessment of overall model fit: the χ^2^ test, the root mean square error of approximation (RMSEA; acceptable fit = < .08, good fit = < .05), the comparative fit index (CFI; acceptable fit = .95–97, good fit = > .97), and the standardized root mean square residual (SRMR; acceptable fit = .05–.10, good fit = < .05; Schermelleh-Engel et al., 2003). Models were compared using the overall model fit indices, the Akaike information criterion (AIC) and Bayesian information criterion (BIC), and the Akaike weights (Wagenmakers & Farrell, 2004).

##### Measurement invariance

Changes in the CFI (ΔCFI) were used to test for measurement invariance (Cheung & Rensvold, 2002). We constrained factor loadings, intercepts, and error terms in that sequence across time points (Widaman et al., 2010). For inferences about changes in factor means over time, intercepts must be temporally invariant (i.e., strong factorial invariance; Meredith, 1993; but see Widaman et al., 2010). When strong invariance was violated, we relaxed intercept constraints for each noninvariant factor separately. Item intercepts were freed sequentially, starting from the item with the largest modification index, until partial invariance was achieved. We compared the results from the fully invariant models with those from the partially invariant models to test the practical significance of assuming strong invariance (Widaman et al., 2010).

##### Exploratory analyses

First, to test the hypothesis that the p factor solely influences its own change, we estimated a model that allows change scores to freely correlate with each other over time. We then used a likelihood ratio test to compare it with the common-cause model with covariances constrained to zero. Second, to assess the impact of gender differences on individual differences in change processes, we added gender as a covariate of latent psychopathology factors at T1 in both models.

### Data Set 2: SHARE

#### Participants

The data were acquired from SHARE. SHARE is a European multinational longitudinal project. The study population consists of all persons who were 50 years or older in 2004 and had their regular residency at a SHARE country during sampling and partners living in the same house regardless of age. Our sample consists of 6,443 persons who had at least one measure of interest (i.e., one item on the EURO-D scale) through the five waves of interest (Waves 1, 2, 4, 5, and 6). Each measurement wave is at a 2-year distance from its predecessor, except Wave 4, which is 4 years apart from Wave 2. All missing data (5.31%) were dealt with using FIML (Enders & Bandalos, 2001). Our age range covers middle adulthood to old age (34–91 years at Wave 1; *M* = 61.5 years, *SD* = 8.3). The sample is ethnically diverse and includes participants from 29 European countries; the Italian majority represents 13.9% of the sample, followed by Sweden (10.9%) and France (10.8%). Of the sample, 57% identified as female, and 43% identified as male. On average, people in our sample completed 10.2 years of education (*SD* = 4.5). For further information regarding data collection and sample characteristics, we refer the reader to the SHARE website (http://www.share-project.org).

#### Measures

We analyzed the EURO-D scale to assess symptoms of MDD (Prince et al., 1999). The measured symptoms are depression, pessimism, suicidality, guilt, sleep, interest, irritability, appetite, fatigue, concentration, enjoyment, and tearfulness. All items assess for prevalence in the last month. Each symptom is measured using one item on a binary scale (0 = not present, 1 = present). Thus, the total score is measured on an ordinal scale with a maximum score of 12. Some items were reverse-coded, so we (re)coded all items so that 1 indicates the presence of symptoms and 0 indicates their absence. Readers interested in the psychometric properties of the EURO-D scale are referred to Prince et al. (1999) and Larraga et al. (2006).

##### Item parceling

**Fig. 3.**
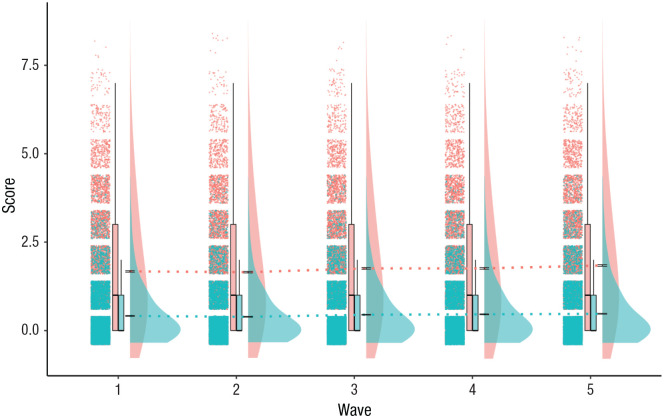
Rain-cloud plot of depression items in the two parcels extracted from the Survey of Health, Ageing and Retirement in Europe (SHARE) data. The first parcel, representing the “affective suffering” construct, included the items of sadness, suicidality, guilt, sleeplessness, irritability, appetite, fatigue, and tearfulness (maximum score = 8). The second parcel, representing the “motivation” construct, included the items of pessimism, interest, concentration, and enjoyment (maximum score = 4). Density plots represent the prevalence of affective suffering and motivation items at each time point such that a higher score indicates higher prevalence of items in the sample. The black horizontal lines at the base of the density plots (approximately at the midpoint) represent the standard error of the mean. The dashed colored lines passing through subsequent waves display changes in the mean prevalence of symptoms over time.

To aid distributional assumptions and the tractability of the structural equation model, we allocated the binary EURO-D symptom items to parcels (Bandalos, 2002; Hau & Marsh, 2004; MacCallum et al., 1999; Matsunaga, 2008; Nunnally, 1978). The two parcels we created mirror the two factors identified in previous psychometric analyses using the EURO-D scale (Castro-Costa et al., 2008; Guerra et al., 2015; Prince et al., 1999). The first parcel representing the “affective suffering” construct included the items of sadness, suicidality, guilt, sleeplessness, irritability, appetite, fatigue, and tearfulness. The second parcel representing the “motivation” construct included the items of pessimism, interest, concentration, and enjoyment. Both parcels were assumed to be continuous (Fig. 3).

#### Statistical analyses (Data Set 2: SHARE)

Below we describe the specification, estimation, and assessment of the models reflecting the two theories. First, we describe the specification of the measurement models and the structural models. Second, we describe how we estimated and fit the models to our data. Measurement invariance was assessed in the same way across data sets.

##### Common cause

**Fig. 4.**
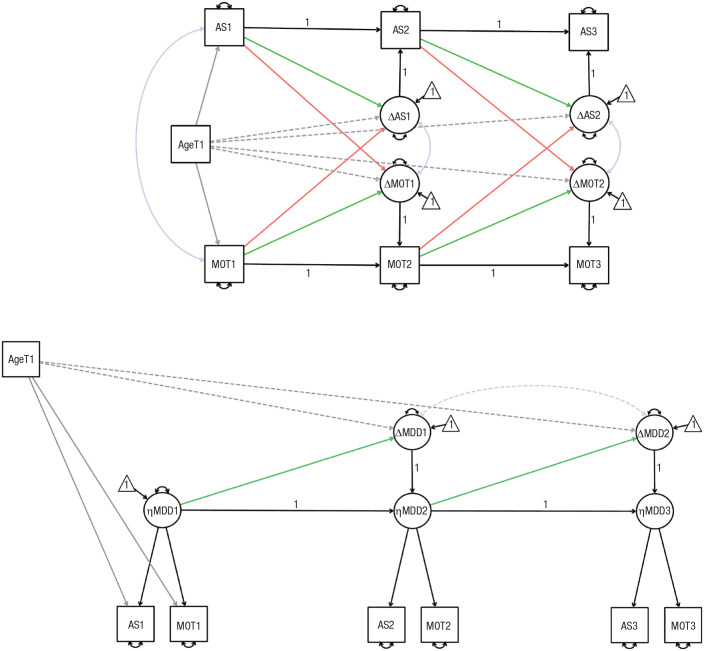
Illustration of dynamic-mutualism model (top) and common-cause model (bottom) for Survey of Health, Ageing and Retirement in Europe (SHARE) data set. Circles indicate latent variables, rectangles indicate parcels, and triangles indicate intercepts. Double-headed arrows indicate covariances (purple) and variances (black). Dashed lines indicate parameters that were included only in exploratory analyses. Single-headed arrows denote regressions. Green and orange single-headed arrows represent the focal parameters for our model comparison. Green single-headed arrows indicate self-feedback parameters (β). Orange single-headed arrows indicate coupling parameters (γ). Gray single-headed arrows indicate associations with age at Time 1 (T1). A “1” shows that the parameter has been constrained to 1. The illustration depicts only three out of five waves (both models) and does not depict covariances between change scores across time (dynamic-mutualism model only) for visual clarity.

Depression is the mechanism that drives development over the assessed time span. Each person’s developmental trajectory is created through the accumulation of latent changes in depression over time. Scores on the two parcel indicators, affective suffering and motivation, are directly caused by changes in depression. This causal structure is modeled using a one-factor confirmatory factor model (Fig. 4). Thus, the mechanism of change operates at the latent level of depression, which causes changes in parcel scores. A univariate LCS model is used to specify the change process at the level of depression. Change in depression at each time point is influenced by two factors. The first is a self-feedback parameter (ϕ, green arrow in Fig. 4), which relates the rate of change in the depression at time *t* to the level of depression at the previous time point. A positive self-feedback parameter reflects accelerated growth, and negative self-feedback is indicative of dampening or statistical artifacts, such as regression to the mean. The second is the indirect effect of prior changes that flow through the fixed-unit paths. This causal flow can be observed by following the fixed-unit paths in Figure 2 (e.g., MDD_*t*2_ = 1 × ΔMDD_*t*2_ → MDD_*t*3_ = 1 × MDD_*t*2_; McArdle, 2009). The common-cause theory predicts that depression is the sole determinant of its change. Thus, we expect the residual change-score covariance to be null. Moreover, we would expect age to have no effect on change scores once self-feedback is taken into account. Self-feedback parameters were freely estimated across waves. This was done to allow the depression factor to have a quantitively different association with its own change across development. We estimated the mean and variance of the LCSs at each wave. The mean and variance of the depression factor were estimated at T1 and equality constrained over time so that any actual change is reflected as change scores. We allowed residual terms to covary between time points for each observed variable with itself to allow indicator-specific variance (Kievit et al., 2017). Age at T1 was included as a covariate to control for the influence of age on the baseline score of the depression factor. We imposed measurement invariance over time. The common-cause model predicts that depression will be invariant over time, reflecting no qualitative differences in the mechanism captured by the latent depression factor across the assessed developmental period.

##### Measurement model for dynamic-mutualism theory

A measurement model was not specified for the dynamic-mutualism model because the structural model specified direct interrelations between the two parcels. Parcels can be seen as latent factors representing the dimension of affective suffering and motivation with equal weights across all their indicators.

##### Structural model for dynamic-mutualism theory

In the dynamic-mutualism model, there is no depression factor that causes people’s observed psychopathology. The development of psychopathology is driven by changes in the dimensions of affective suffering and motivation. Thus, each person’s developmental trajectory is created through the accumulation of changes in the two dimensions of affective suffering and motivation. Change in the dimensions of affective suffering and motivation explain change in the prevalence of their constituent symptoms. We used bivariate LCS models to specify the process of change at the level of the two dimensions. Change in each dimension at each time point is determined by three factors. The first is a self-feedback parameter (ϕ, green arrow in Fig. 4), which relates the rate of change in a given dimension (e.g., motivation) at time *t* to the level of the same dimension (motivation again) at the previous time point. A positive self-feedback parameter reflects accelerated growth, whereas negative self-feedback is indicative of dampening or statistical artifacts such as regression to the mean. The second is coupling parameters (γ), which reflect the influence of the level in a given dimension (e.g., motivation) at the preceding time *t* − 1 on the rate of change in the other at time *t* (e.g., affective suffering). The coupling parameters reflect the core mechanism of dynamic mutualism (i.e., causal interactions between distinct dimensions of psychopathology) and are related to the M matrix in the mutualism model (Hofman et al., 2018; van der Maas et al., 2006). The third is the indirect effect of prior changes that flow through the fixed-unit paths. This causal flow can be observed by following the fixed-unit paths in Figure 2 (e.g., MOT_*t*2_ = 1 × ΔMOT_*t*2_→ MOT_*t*3_ = 1 × MOT_*t*2_; McArdle, 2009). The mutualism model predicts predominantly positive bidirectionality between dimensions (i.e., most coupling parameters should be positive). If the tempo of sampling does not match the temporal unfolding of the developmental mechanism but positive causal interactions are still the dominant driving force of development in between the assessments, the coupling parameters should be positive, but the effect sizes will be inflated. We estimated the mean and variance of the LCSs at each wave. The mean and variance of affective suffering and motivation were estimated at T1 and equality constrained over time so that any actual change is reflected as change scores. Both self-feedback parameters and coupling parameters were freely estimated across time points to allow the association between the current state and subsequent change to quantitively differ across development. Affective suffering and motivation were allowed to correlate at T1. To allow indicator-specific variance, we allowed residual terms to covary between time points for each observed variable with itself (Kievit et al., 2017). Dynamic-mutualism theory allows for unmodeled symptoms/disorders to affect change in psychopathology; therefore, latent change factors were allowed to correlate within and between time points. Age at T1 was included as a covariate to control for the influence of age on baseline affective-suffering and motivation scores.

##### Exploratory statistical analyses

We extended our models in three exploratory analyses. First, to test the hypothesis that the depression factor was the sole influence of its change, we allowed the change scores of the common-cause model to correlate with each other across time. Second, to test whether the rate of developmental change in psychopathology was solely explained by the dynamics in the two models, we estimated the direct effect of age on change scores (Kievit et al., 2017). We conducted likelihood ratio tests to assess whether the added parameters significantly improved the models. Third, to assess the impact of gender differences on individual differences in change processes, we added gender as a covariate of latent psychopathology factors at T1 in both models.

### Preregistration

All confirmatory analyses were conducted according to our preregistered plan (https://osf.io/yezgt); deviations are reported in Table S8 in the Supplemental Material. Deviations were based on model-convergence issues, or knowledge about the data we did not possess before preregistration, and not based on results.

## Results

### Study 1: p factor of psychopathology

**Fig. 5.**
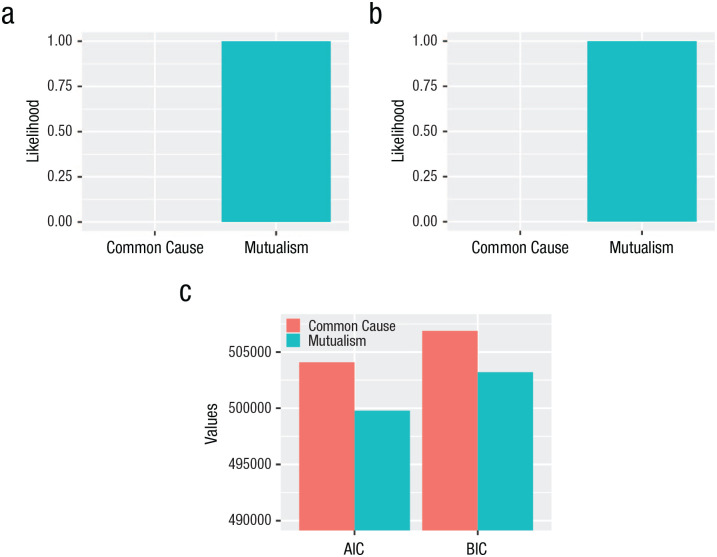
Normalized probabilities indicated by (a) Akaike weights, (b) Schwarz weights, and (c) information criterion (AIC) and Bayesian information criterion (BIC) for each model.

We first specified and fitted the LCS models (structural-equation-modeling framework) representing the common-cause theory and the dynamic-mutualism theory of psychopathology to the z-proso data. We examined factors loadings and measurement invariance. Thereafter, we compared the ability of the two (nonnested) theoretical models to explain the development of the p factor based on a preregistered set of fit indices (Table 1, Fig. 5).

**Table 1.** Model Comparison Fit Statistics for z-Proso Models

Model	χ^2^	*df*	RMSEA	CFI	SRMR	AIC	BIC
Common cause	35,194.326	13,835	.034 [.034, .035]	.749	.108	504,085.954	506,890.261
Mutualism	31,201.888	13,716	.031 [.031, .032]	.795	.067	499,782.029	503,217.173
Exploratory common cause^a^	35,187.979	13,832	.034 [.034, .035]	.749	.108	504,082.549	506,902.760

Note: z-Proso = Zurich Project on the Social Development of Children and Youths; RMSEA = root mean square error of approximation; CFI = comparative fit index; SRMR = standardized root mean square residual; AIC = Akaike information criterion; BIC = Bayesian information criterion.

a Common-cause model with residual change score covariances. Numbers in brackets indicate the 90% confidence interval for the RMSEA.

#### Factor specification

Standardized factor loadings for the 42 symptom items were moderate to strong (see Table S13 in the Supplemental Material).

#### Measurement invariance

We determined measurement invariance was violated when ΔCFI exceeded the cutoff of .01 (Cheung & Rensvold, 2002). First, we imposed weak measurement invariance, which led to a negligible drop in fit for both models according to the proposed cutoff (common cause: ΔCFI = .010; mutualism: ΔCFI = .002; Cheung & Rensvold, 2002). Second, we constrained intercepts to be equal over time, which led to a substantial drop in fit for both models (common cause: ΔCFI = .028; mutualism: ΔCFI = .029). Note that the violation of temporal invariance is expected under certain conditions of mutualism theory but cannot be considered conclusive evidence for the theory because numerous alternative causes of noninvariance exist (e.g., response-shift bias; Fokkema et al., 2013). Next, we compared the results from the fully invariant models with the partially invariant models to test the practical significance of assuming strong measurement invariance (Widaman et al., 2010).

#### Model comparison

The dynamic-mutualism model fit better according to all preregistered fit statistics. Both models fit the data well according to all fit indices except the CFI (Table 1). This conclusion is mirrored by the information criteria (AIC and BIC; Fig. 5), which were used to take into account complexity in terms of the number of freely estimated parameters (the mutualism model has more parameters and is therefore more likely to describe the data well). The Akaike weights, which quantify the conditional probability that a model is the most correct (Wagenmakers & Farrell, 2004), show that given our data and the candidate models assessed, the mutualism model is 99.99% likely to be the better model (Fig. 5). The partially invariant models mirrored these conclusions (see Table S1 in the Supplemental Material). Because model comparisons exhibited the same patterns under both the fully and partially invariant models, the violation of temporal invariance is exceedingly unlikely to affect our core inferences (Widaman et al., 2010, p.13).

#### Model parameters

We closely examined the parameters of both models (Fig. 2). We interpreted the significance of model parameters on the basis of statistical significance and the effect-size guidelines by Gignac and Szodorai (2016). Note that prosociality was reverse-coded so that higher scores indicate lower prosociality. Higher scores on all other dimensions indicate higher severity of psychopathology (e.g., an ADHD score of 5 means more severe ADHD than a score of 4).

##### Common-cause model

All regression parameters are presented in Table S15 in the Supplemental Material. First, we found substantial change in the p factor at each time point. Average change ranged from *b* = 0.51 to 0.58. We also found considerable interindividual variability in the rate of change of the p factor (see Table S16 in the Supplemental Material). Second, at Wave 2, the p factor did not explain its own change (i.e., a negligible self-feedback effect; *b* = −0.01, *SE* = 0.07, β = −0.01^1^). The evidence for the ability of the p factor to explain its own change at Wave 3 was mixed. The self-feedback effect at Wave 3 was weak according to Gignac and Szodorai (2016) and not statistically significant (*b* = −0.07, *SE* = 0.04, β = −0.12). The p factor explained its own change at Wave 4. Higher levels of the p factor predicted a lower rate of change in p, with a moderate self-feedback effect (*b* = −0.157, *SE* = 0.04, β = −0.27). These results are not in line with the p factor as a stable, generalized, liability for mental illness because only one self-feedback effect can be labeled insignificant according to our evaluation criteria.

##### Mutualism model

All regression parameters are reported in Table S2 in the Supplemental Material. Psychopathology domains were significantly positively correlated at baseline, except for ADHD and prosociality, which were not significantly related (*b* = −0.03, *SE* = 0.019, *p* = .15). Persons varied substantially in their rate of change in all domains (see Table S3 in the Supplemental Material).

As is often the case in these models, higher scores in any domain were significantly negatively associated with change in the same domain over time (apart from the association between internalizing at Wave 3 and change at Wave 4, which was moderate but not significant). The self-feedback parameters (green arrows in Fig. 2) ranged from moderate to strong (*b*s = −0.168 to −0.464, *SE*s = 0.029 to 0.109, βs = −0.197 to −0.550). This could indicate that individuals’ level of psychopathology in a certain domain (e.g., internalizing) is negatively related with their degree of change in that domain over time (i.e., a dampening effect). Alternatively, the negative self-feedback effects could be due to regression to the mean, ceiling effects, or a combination of statistical artifacts and substantive mechanisms.

Twenty-one coupling parameters (orange arrows in Fig. 2) were positive, and 15 were negative. Thirty-one coupling effects were not significant. Thus, the scores of most dimensions across most time points were not substantively associated with change in most dimensions at the subsequent time point. In the five cases in which coupling effects were significant, three were positive, and two were negative. The externalizing dimension was significantly associated only with change in one domain at one time point. Higher scores in the externalizing domain at T1 were weakly and negatively associated with change in internalizing at T2 (*b* = −0.146, *SE* = 0.050, β = −0.125). Prosociality influenced the internalizing and externalizing dimensions, although not across all waves. Higher prosociality scores at T3 were moderately and positively associated with change in internalizing at T4 (*b* = 0.205, *SE* = 0.101, β = 0.213). Higher scores in prosociality at T1 were positively associated with change in externalizing at T2 (*b* = 0.059, *SE* = 0.022, β = 0.092). However, the size of this effect did not meet the effect-size cutoff of 0.10 (Gignac & Szodorai, 2016). ADHD scores were not significantly associated with change in any dimension. Finally, higher scores on the internalizing dimension at T1 were positively and moderately associated with change in ADHD at T2 (*b* = 0.128, *SE* = 0.038, β = 0.146). Internalizing at T1 was also negatively related to change in prosociality at T2, but the size of this effect was negligible (*b* = −0.064, *SE* = 0.032, β = −0.067).

#### Exploratory analyses

In line with the hypothesis that the p factor influences solely its own change but is not necessarily stable, allowing residual correlations between change scores did not improve the fit of the preregistered common-cause model, Δχ^2^(3) = 6.61, *p* = .09. Adding gender differences as a covariate in our models did not alter our main conclusions (for detailed results, see Table S17–S19 in the Supplemental Material).

### Study 2: major depression symptoms

**Fig. 6.**
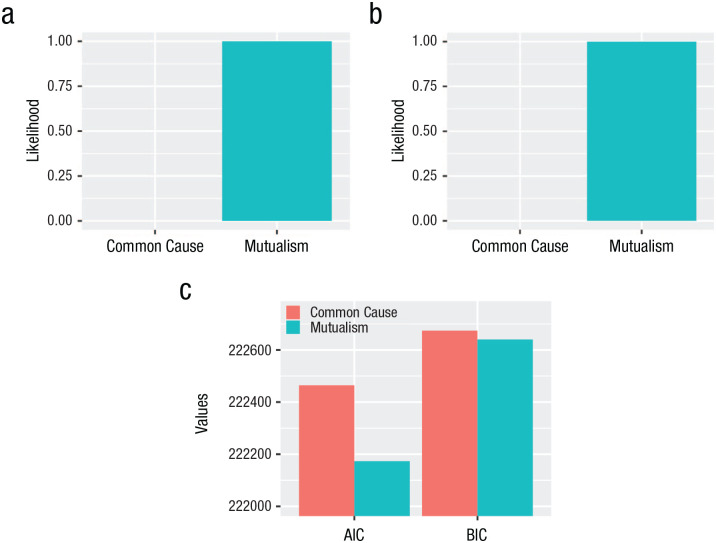
Normalized probabilities indicated by (a) Akaike weights, (b) Schwarz weights, and (c) Akaike information criterion (AIC) and Bayesian information criterion (BIC) for each model.

We specified and fitted latent change-score models (structural-equation-modeling framework) representing the common-cause theory and the mutualism theory to the SHARE data (Fig. 4). We examined measurement invariance for the common-cause model. The mutualism model did not include latent variables, and therefore, we could not examine measurement invariance. Next, we compared the ability of the two (nonnested) theoretical models to explain change in symptoms included in major depression based on a preregistered set of fit indices (Table 2, Fig. 6).

**Table 2.** Model Comparison Fit Statistics for SHARE Data

Model	χ^2^	*df*	RMSEA	CFI	SRMR	AIC	BIC
Common cause	505.073	46	.043 [.040, .047]	.966	.034	222,464.586	222,674.480
Mutualism	232.744	8	.068 [.060, .075]	.984	.029	222,173.390	222,640.572

Note: SHARE = Survey of Health, Ageing and Retirement in Europe; RMSEA = root mean square error of approximation; CFI = comparative fit index; SRMR = standardized root mean square residual; AIC = Akaike information criterion; BIC = Bayesian information criterion.Numbers in brackets indicate the 90% confidence interval for the RMSEA.

#### Measurement invariance

Temporal invariance was not violated for the common-cause model (weak invariance ΔCFI = 0.000; strong invariance ΔCFI = 0.000; Cheung & Rensvold, 2002).

#### Model comparison

We examined fit indices to determine which model better explains change in major depression. One of the preregistered fit indices showed preferential support for the dynamic-mutualism model (CFI), whereas another (RMSEA) supported the common-cause model (Table 2). The information criteria both supported the dynamic mutualism model (AIC and BIC; Fig. 6). Both the Akaike and Schwarz weights show that the dynamic mutualism model is 99.99% more likely to be the better model given our data and the set of assessed models (Fig. 6).

#### Model parameters

We examined the parameters of both models. We interpreted the significance of model parameters on the basis of statistical significance and the effect-size guidelines by Gignac and Szodorai (2016). Higher scores on the parcel indicators (affective suffering and motivation) indicate greater severity of psychopathology.

##### Common-cause model

All regression parameters are presented in Table S4 in the Supplemental Material. We found considerable interindividual variability in the rate of change of the depression factor (Table S5 in the Supplemental Material). Higher age at baseline predicted higher depression-factor scores at baseline, as evidenced by the substantial drop in fit after we fixed the effect of age at baseline on the depression factor at baseline to 0, Δχ^2^(1) = 6.73, *p* = .01. Across all measurement waves, higher scores on the depression factor were negatively associated with change in depression at the next time point. The negative self-feedback effects ranged from weak to strong (*b*s = −0.090 to −0.271, *SE*s = 0.026 to 0.040, βs = −0.142 to −0.455).

##### Dynamic-mutualism model

All regression parameters are reported in Table S6 in the Supplemental Material. We found evidence for individual differences in the rate of change in both domains (Table S5 in the Supplemental Material). Age at baseline was positively associated with the motivation parcel and negatively associated with the affective-suffering parcel at baseline, Δχ^2^(2) = 28.14, *p* < .001. Constraining residual-change score covariances to zero substantially affected model fit, Δχ^2^(28) = 3420.3, *p* < .001. This supports the existence of unmeasured influences on change in the two domains and/or temporal mismatch between measurement and the natural pace of change (Hofman et al., 2018).

##### Self-feedback effects

The affective-suffering domain significantly influenced its own change across three of the four measurement waves. The significant self-feedback effects were negative and ranged from weak to strong (*b*s = −0.099 to −0.504, *SE*s = 0.013 to 0.043, βs = −0.097 to −0.517). The exception was the association between affective suffering at T4 and change in affective suffering at T5, which was not significant (*b* = 0.015, *SE* = 0.052, β = 0.016). The motivation domain was significantly associated only with its own change at one time point. Higher scores on motivation at T1 were negatively associated with change in the motivation domain at T2 (*b* = −0. 727, *SE* = 0.018, β = −0.642). Self-feedback parameters reflect a combination of effects, including regression to the mean, which may be responsible for the size of the effect (Kievit et al., 2017).

##### Coupling effects

Most coupling effects indicated that scores on the motivation domain were not significantly associated with change in the affective suffering domain and vice versa. Six coupling parameters were positive, and two were negative. Only two coupling effects were significant, and both were positive. Higher scores on motivation at T1 were positively related to change in affective suffering at T2 (*b* = 0.140, *SE* = 0.031, β = 0.064). The size of this effect was, however, negligible. Higher scores in affective suffering at T1 were weakly and positively associated with change in the motivation domain at T2 (*b* = 0.057, *SE* = 0.006, β = 0.113).

#### Exploratory analyses

Fit statistics for the exploratory models are presented in Table S7 in the Supplemental Material. First, our results did not support the hypothesis that the depression factor is solely responsible for its own change because allowing change scores to covary over time led to substantial improvement in model fit, Δχ^2^(6) = 95.58, *p* < .001. Second, both the dynamic-mutualism model and the common-cause model failed to capture all age-related dynamics because allowing age to directly affect change scores led to a significant improvement in model fit; common cause: Δχ^2^(4) = 161.73, *p* < .001; mutualism: Δχ^2^(2) = 345.61, *p* < .001. Third, adding gender differences as a covariate in our models did not significantly alter our main conclusions (for detailed results, see Table S20–S22 in the Supplemental Material).

## Discussion

We directly compared the ability of the common-cause theory and the dynamic-mutualism theory to explain the development of individual differences in two domains of psychopathology at two different developmental periods using two large developmental cohorts—the p factor from early to late adolescence (*N* = 1,482) and major depression in middle adulthood and old age (*N* = 6,443). We did so by translating these two theories into statistical models that impose theory-consistent assumptions on the data. Our findings strongly question the idea that a single dimension or coupling among its constituent parts can fully explain the development of the p factor or major depression. We summarize the main results from each data set, followed by the implications of our results for the study of psychopathology and recommendations for future work.

In our first investigation using the z-proso data, we compared the ability of our models to explain the development of the statistical p factor from childhood to early adolescence. Both our statistical models performed poorly relative to the baseline model, but the models performed well compared with a saturated model (i.e., poor CFI, good RMSEA). The mutualism model fit better than the common-cause model. However, the model’s parameters did not corroborate a core prediction derived from dynamic-mutualism theory: predominantly positive bidirectionality. That is, for the positive manifold to emerge from mutualistic coupling, *most* associations between causal agents should be positive (van der Maas et al., 2006), whereas the coupling effects in our model were mostly negligible, and approximately half were negative. One explanation is that a mutualistic account of psychopathology does not hold. Alternatively, the result may be due to a mismatch between the temporal pace of causal relations across dimensions and the time lags in our sample. For example, the causal connection between a sleepless night and fatigue may happen overnight but will not be captured by the association between sleeplessness and fatigue a year apart. This mismatch does not exist because of a poor statistical representation of the theory but, rather, shows the breadth of interpretative freedom the dynamic-mutualism theory of psychopathology currently allows, calling for more formal theoretical developments (Borsboom et al., 2021; Fried, 2020; Robinaugh et al., 2021).

The psychological dimensions assessed (e.g., internalizing) were not temporally invariant. The lack of temporal invariance could be due to a measurement artifact, or it may be an indication that the causes of the shared variance among symptoms fluctuate over time. The latter interpretation is in line with the hypothesis that the structure of psychopathology is not developmentally stable and may not be readily reducible to any simple structure (Wittchen et al., 2009). Only a few other studies have examined the longitudinal invariance of the p factor with mixed results (Castellanos-Ryan et al., 2016; Gluschkoff et al., 2019; Snyder et al., 2017). The developmental stability of individual differences in the statistical p factor, however, has been supported by numerous studies (Castellanos-Ryan et al., 2016; Greene & Eaton, 2017; McElroy et al., 2018; Snyder et al., 2017), except for research using the z-proso data set, which reported much lower stability indices (Murray et al., 2016). At the moment, the evidence supports the hypothesis that people who experience relatively more severe psychopathology symptoms early on tend to also experience relatively more severe psychopathology later in their life (i.e., stable rank-order of individual differences). In contrast, more work is needed to make reliable conclusions about the longitudinal invariance of the p factor. Future work should examine (a) the time span of invariance in the p factor, (b) to what extent and in what ways different item sets might confer different results, and (c) whether conventional fit-index cutoffs are appropriate for determining the temporal invariance of higher-order models typically used to quantitatively structure psychopathology.

The evidence from our first study suggests that the generative process underpinning the p factor is multifactorial and fluctuates across the life span. This goes against the idea that the p factor reflects “a quantitatively distributed, stable, generalized liability to develop any and all forms of psychopathology across the life course” (Caspi & Moffitt, 2018, p. 840). If the p factor exists as a stable generalized vulnerability factor, we hypothesize that it is responsible for only a small portion of the variance summarized by its statistical counterpart. Likewise, our results do not support reciprocal causal interactions among distinct dimensions of psychopathology as a dominant explanation for the variance summarized by the statistical p factor.

In our second investigation using the SHARE data, we could not clearly ascertain which model better explained change in major depression during the developmental periods of middle adulthood and old age. The dynamic-mutualism model had more explanatory power while explicitly weighing parsimony using information criteria and performed better relative to the baseline model (higher CFI). Conversely, the common-cause model performed better relative to a saturated model (lower RMSEA). The direction and size of coupling effects was, again, not in line with a mutualistic account of psychopathology. Crucially, neither theory could fully explain age-related changes in depression symptoms, and exploratory analyses supported the idea that influential unmeasured factors (e.g., life events, onset of developmentally specific biological processes) were missing from our models. For example, the number of drug-using friends is one of the best predictors of adolescent substance abuse (Cousijn et al., 2018), but neither theory explicitly defines the functional relations between interpersonal processes, symptoms, and/or neurobiological factors.

In sum, our results are in line with the unanimous conclusion of past longitudinal studies: Neither theory can fully explain the development of psychopathology (Greene & Eaton, 2017; McArdle et al., 2000; McElroy et al., 2018; Murray et al., 2016; Snyder et al., 2017). Hence, it may be better to start looking at what percentage of variance in the developmental dynamics of psychopathology each theory can explain. Hybrid models in which common causes and dynamic mutualism come together may provide promising multicausal explanations for the development of psychopathology (Fried & Cramer, 2017; for an example in general intelligence, see van der Maas et al., 2017). For instance, general vulnerability to psychopathology may reinforce causal interactions between symptoms by lowering their activation threshold. This could increase the probability that symptoms are caused by environmental events and other symptoms. In turn, symptoms may exacerbate this general vulnerability (e.g., effect of sleep on stress-response system; Koss & Gunnar, 2018; Ly et al., 2015). Alternatively, specific types of risk factors may lead to specific disorders and interactions among the presenting symptoms, and environmental factors may lead to comorbidity. This would be consistent with an interpretation of the p factor as an amalgamation of distinct causes (Krueger et al., 2018; Watts et al., 2019) that may cohere because of the causal interrelations among their outcomes. Both scenarios, and multiple others (for more examples, see Fried & Cramer, 2017) in which latent pathophysiology coexists and possibly interacts with mechanisms at the level of manifest psychopathology, may explain the development of the positive manifold. Moreover, different mechanisms and their interactions may lead to the development of different disorders, and the same disorder may be explained by several mechanisms (Borsboom et al., 2019). Thus, embracing a multicausal framework is likely the only suitable candidate model to make meaningful progress in understanding, predicting, and treating mental illness because it acknowledges the massively multifactorial nature of psychopathology (Fried & Robinaugh, 2020; Kendler, 2019).

Our conclusions need to be considered alongside our limitations. Readers who disagree with our implementation of either theory are encouraged to propose distinct, dynamic, precisely formalized alternatives (for arguments regarding the importance of formal models, see Borsboom et al., 2021; Fried, 2020). Readers who wish to build on our models can start with the following limitations.

First, in the dynamic-mutualism models, we specified causal interrelations between all the constituent parts of the model. However, it is more likely that a psychopathology network includes both direct and indirect associations, including mediation by other symptoms and environmental/interpersonal factors. Future research could directly compare mutualism models that include different causal paths to improve our understanding of causal associations and improve the specificity of theory; such work should happen in both data-driven and theory-driven ways.

Second, we evaluated the practical significance of the parameters in our models on the basis of their statistical significance and the effect-size guidelines by Gignac and Szodorai (2016). It is, however, not given that an effect we deemed significant is strong enough to causally alter a distinct domain of psychopathology or meaningfully contribute to an accumulative process to change psychopathology over time. Our understanding of dynamic mutualism would benefit from future simulation and experimental work that aims to identify sufficient effect sizes that would support causal interactions under mutualism.

Third, we specified associations among variables over the durations provided by our samples (i.e., years apart). However, we do not know whether causal mechanisms unfold over this time span—certain canonical examples of causal interactions between symptoms (e.g., poor sleep causing feelings of fatigue) certainly operate at much shorter timescales. Future studies should aim to elucidate the chronometry of causal associations among symptoms and/or disorders. Ecological momentary assessment (EMA) could be a useful tool for exploring more fine-grained processes that may form the building blocks of psychopathology (Wichers, 2014). EMA data can also overcome problems with well-documented biases that accompany asking participants to recollect their behavior within a past window of time (Onnela, 2021).

Fourth, we restricted the theoretical space to two influential theories of psychopathology. We believe that these mechanisms are active components across a large breadth of mental illness. However, this does not mean that psychopathology is necessarily restricted to an iteration of these two alternatives. Formalizing alternative theories and comparing them with common-cause theory and dynamic-mutualism theory is a viable endeavor that some readers may be inclined to follow.

Fifth, we used item parcels to normalize SHARE data, but it is questionable whether the parcels reflect substantive constructs. Although the content of the parcels was based on constructs that have been recurrently identified in prior studies using the EURO-D scale (Castro-Costa et al., 2008; Guerra et al., 2015; Prince et al., 1999), the constructs are quantitative creations, and the theoretical coherence between the items is questionable. This could lead to symptoms that exhibit causal interactions being bulked in a parcel, leading to a weak test of dynamic mutualism. In addition, parcels attribute equal weight to all items, which can lead to bias proportional to the difference between the item coefficient in the true model and the weight specified by the sum score (Bollen & Bauldry, 2011). We would ideally use multiitem continuous measures of individual symptoms to create a dynamic-mutualism model that specifies interrelations among symptoms directly. Currently, systematic empirical evidence for causally related symptoms in the domain of depression is lacking because of the novelty of the field and methodological differences in studies (e.g., lags and item content) across studies using network statistics. This is compounded by the lack of theoretical rationale for specific symptom-symptom interactions, making identification of specific causal interactions a pressing issue for future research.

Sixth, for the means of testing the ontology of the p factor, we assumed that broad transdiagnostic dimensions are adequately described using reflective latent variables. However, it may be that these dimensions are, at least partly, the product of mutualistic coupling among symptoms. That is, the substantive meaning of the statistical dimensions underlying the p factor also remains an empirical question. Future work would benefit from assessing the mechanisms that explain the coherence among symptoms starting from the lowest level of abstraction.

Seventh, the findings of the present study are limited to the populations assessed in the analyzed samples and to the measured dimensions of psychopathology. Research focusing on different developmental periods could assess homogeneity in psychological processes throughout development. In our study, we found that model-fit statistics supported mutualism over the common-cause theory in the younger cohort but not the older cohort. A speculative interpretation for this difference in findings could be that the decrease in plasticity that accompanies older age is a cause of differences in the involvement of developmental mechanisms. For instance, dynamic mutualism could be a poor explanation of psychopathology in the older sample owing to well-documented (Kühn & Lindenberger, 2016) decreases in plasticity that accompany aging. This decrease in malleability could make learning associations between behaviors harder, making dynamic mutualism a less involved determinant of psychopathology. However, the present evidence does not allow us to make strong claims about the interplay between neural and behavioral mechanisms. The emergence of large, rich longitudinal cohorts such as ABCD (Volkow et al., 2018) will allow for more robust investigations of such hypotheses in the future.

Finally, we added gender as a covariate only in exploratory analyses to assess its impact on differences in the developmental mechanisms we tested. In light of the evidence on sex and gender differences in psychopathology (e.g., Hartung & Lefler, 2019; Rutter et al., 2003), it would be interesting for future studies to conduct a more detailed assessment of the impact of gender and sex-related variables as a source of differences in the development of psychopathology.

It is time to acknowledge that if researchers want to make sense of the complex systems of psychopathology, they need longitudinal data and formal models to get there. Panel data allowed us to peer into the dynamics of psychopathology, but we were still confined to the study of group-level dynamics of individual differences. The causal mechanisms that drive psychopathology operate at an unknown timescale and in the individual. Thus, data of high temporal resolution are required to elucidate the dynamics that govern a person’s mental health. In turn, new insights from detailed data will require a greater reliance on formal models (Borsboom et al., 2021; Lövdén et al., 2020; Robinaugh et al., 2021). Formal models have the added benefit of allowing researchers to build theories iteratively and independently by limiting the interpretative freedom allowed by verbal theory (Fried, 2020; Smaldino, 2017). But to build useful theories as a community, researchers need to openly share their work so that others can improve it. Our code is available on https://osf.io/a4ywe/?view_only=498f5640c18847bea3ac6a9b0b596821, and we encourage others to build on it so that the field can progressively edge itself closer to a better understanding of mental illness.

## Supplemental Material

sj-docx-1-cpx-10.1177_21677026231162814 – Supplemental material for Common Cause Versus Dynamic Mutualism: An Empirical Comparison of Two Theories of Psychopathology in Two Large Longitudinal Cohorts Supplemental material, sj-docx-1-cpx-10.1177_21677026231162814 for Common Cause Versus Dynamic Mutualism: An Empirical Comparison of Two Theories of Psychopathology in Two Large Longitudinal Cohorts by Michael E. Aristodemou, Rogier A. Kievit, Aja L. Murray, Manuel Eisner, Denis Ribeaud and Eiko I. Fried in Clinical Psychological Science

sj-docx-10-cpx-10.1177_21677026231162814 – Supplemental material for Common Cause Versus Dynamic Mutualism: An Empirical Comparison of Two Theories of Psychopathology in Two Large Longitudinal Cohorts Supplemental material, sj-docx-10-cpx-10.1177_21677026231162814 for Common Cause Versus Dynamic Mutualism: An Empirical Comparison of Two Theories of Psychopathology in Two Large Longitudinal Cohorts by Michael E. Aristodemou, Rogier A. Kievit, Aja L. Murray, Manuel Eisner, Denis Ribeaud and Eiko I. Fried in Clinical Psychological Science

sj-docx-11-cpx-10.1177_21677026231162814 – Supplemental material for Common Cause Versus Dynamic Mutualism: An Empirical Comparison of Two Theories of Psychopathology in Two Large Longitudinal Cohorts Supplemental material, sj-docx-11-cpx-10.1177_21677026231162814 for Common Cause Versus Dynamic Mutualism: An Empirical Comparison of Two Theories of Psychopathology in Two Large Longitudinal Cohorts by Michael E. Aristodemou, Rogier A. Kievit, Aja L. Murray, Manuel Eisner, Denis Ribeaud and Eiko I. Fried in Clinical Psychological Science

sj-docx-12-cpx-10.1177_21677026231162814 – Supplemental material for Common Cause Versus Dynamic Mutualism: An Empirical Comparison of Two Theories of Psychopathology in Two Large Longitudinal Cohorts Supplemental material, sj-docx-12-cpx-10.1177_21677026231162814 for Common Cause Versus Dynamic Mutualism: An Empirical Comparison of Two Theories of Psychopathology in Two Large Longitudinal Cohorts by Michael E. Aristodemou, Rogier A. Kievit, Aja L. Murray, Manuel Eisner, Denis Ribeaud and Eiko I. Fried in Clinical Psychological Science

sj-docx-13-cpx-10.1177_21677026231162814 – Supplemental material for Common Cause Versus Dynamic Mutualism: An Empirical Comparison of Two Theories of Psychopathology in Two Large Longitudinal Cohorts Supplemental material, sj-docx-13-cpx-10.1177_21677026231162814 for Common Cause Versus Dynamic Mutualism: An Empirical Comparison of Two Theories of Psychopathology in Two Large Longitudinal Cohorts by Michael E. Aristodemou, Rogier A. Kievit, Aja L. Murray, Manuel Eisner, Denis Ribeaud and Eiko I. Fried in Clinical Psychological Science

sj-docx-14-cpx-10.1177_21677026231162814 – Supplemental material for Common Cause Versus Dynamic Mutualism: An Empirical Comparison of Two Theories of Psychopathology in Two Large Longitudinal Cohorts Supplemental material, sj-docx-14-cpx-10.1177_21677026231162814 for Common Cause Versus Dynamic Mutualism: An Empirical Comparison of Two Theories of Psychopathology in Two Large Longitudinal Cohorts by Michael E. Aristodemou, Rogier A. Kievit, Aja L. Murray, Manuel Eisner, Denis Ribeaud and Eiko I. Fried in Clinical Psychological Science

sj-docx-15-cpx-10.1177_21677026231162814 – Supplemental material for Common Cause Versus Dynamic Mutualism: An Empirical Comparison of Two Theories of Psychopathology in Two Large Longitudinal Cohorts Supplemental material, sj-docx-15-cpx-10.1177_21677026231162814 for Common Cause Versus Dynamic Mutualism: An Empirical Comparison of Two Theories of Psychopathology in Two Large Longitudinal Cohorts by Michael E. Aristodemou, Rogier A. Kievit, Aja L. Murray, Manuel Eisner, Denis Ribeaud and Eiko I. Fried in Clinical Psychological Science

sj-docx-16-cpx-10.1177_21677026231162814 – Supplemental material for Common Cause Versus Dynamic Mutualism: An Empirical Comparison of Two Theories of Psychopathology in Two Large Longitudinal Cohorts Supplemental material, sj-docx-16-cpx-10.1177_21677026231162814 for Common Cause Versus Dynamic Mutualism: An Empirical Comparison of Two Theories of Psychopathology in Two Large Longitudinal Cohorts by Michael E. Aristodemou, Rogier A. Kievit, Aja L. Murray, Manuel Eisner, Denis Ribeaud and Eiko I. Fried in Clinical Psychological Science

sj-docx-17-cpx-10.1177_21677026231162814 – Supplemental material for Common Cause Versus Dynamic Mutualism: An Empirical Comparison of Two Theories of Psychopathology in Two Large Longitudinal Cohorts Supplemental material, sj-docx-17-cpx-10.1177_21677026231162814 for Common Cause Versus Dynamic Mutualism: An Empirical Comparison of Two Theories of Psychopathology in Two Large Longitudinal Cohorts by Michael E. Aristodemou, Rogier A. Kievit, Aja L. Murray, Manuel Eisner, Denis Ribeaud and Eiko I. Fried in Clinical Psychological Science

sj-docx-18-cpx-10.1177_21677026231162814 – Supplemental material for Common Cause Versus Dynamic Mutualism: An Empirical Comparison of Two Theories of Psychopathology in Two Large Longitudinal Cohorts Supplemental material, sj-docx-18-cpx-10.1177_21677026231162814 for Common Cause Versus Dynamic Mutualism: An Empirical Comparison of Two Theories of Psychopathology in Two Large Longitudinal Cohorts by Michael E. Aristodemou, Rogier A. Kievit, Aja L. Murray, Manuel Eisner, Denis Ribeaud and Eiko I. Fried in Clinical Psychological Science

sj-docx-19-cpx-10.1177_21677026231162814 – Supplemental material for Common Cause Versus Dynamic Mutualism: An Empirical Comparison of Two Theories of Psychopathology in Two Large Longitudinal Cohorts Supplemental material, sj-docx-19-cpx-10.1177_21677026231162814 for Common Cause Versus Dynamic Mutualism: An Empirical Comparison of Two Theories of Psychopathology in Two Large Longitudinal Cohorts by Michael E. Aristodemou, Rogier A. Kievit, Aja L. Murray, Manuel Eisner, Denis Ribeaud and Eiko I. Fried in Clinical Psychological Science

sj-docx-2-cpx-10.1177_21677026231162814 – Supplemental material for Common Cause Versus Dynamic Mutualism: An Empirical Comparison of Two Theories of Psychopathology in Two Large Longitudinal Cohorts Supplemental material, sj-docx-2-cpx-10.1177_21677026231162814 for Common Cause Versus Dynamic Mutualism: An Empirical Comparison of Two Theories of Psychopathology in Two Large Longitudinal Cohorts by Michael E. Aristodemou, Rogier A. Kievit, Aja L. Murray, Manuel Eisner, Denis Ribeaud and Eiko I. Fried in Clinical Psychological Science

sj-docx-20-cpx-10.1177_21677026231162814 – Supplemental material for Common Cause Versus Dynamic Mutualism: An Empirical Comparison of Two Theories of Psychopathology in Two Large Longitudinal Cohorts Supplemental material, sj-docx-20-cpx-10.1177_21677026231162814 for Common Cause Versus Dynamic Mutualism: An Empirical Comparison of Two Theories of Psychopathology in Two Large Longitudinal Cohorts by Michael E. Aristodemou, Rogier A. Kievit, Aja L. Murray, Manuel Eisner, Denis Ribeaud and Eiko I. Fried in Clinical Psychological Science

sj-docx-21-cpx-10.1177_21677026231162814 – Supplemental material for Common Cause Versus Dynamic Mutualism: An Empirical Comparison of Two Theories of Psychopathology in Two Large Longitudinal Cohorts Supplemental material, sj-docx-21-cpx-10.1177_21677026231162814 for Common Cause Versus Dynamic Mutualism: An Empirical Comparison of Two Theories of Psychopathology in Two Large Longitudinal Cohorts by Michael E. Aristodemou, Rogier A. Kievit, Aja L. Murray, Manuel Eisner, Denis Ribeaud and Eiko I. Fried in Clinical Psychological Science

sj-docx-22-cpx-10.1177_21677026231162814 – Supplemental material for Common Cause Versus Dynamic Mutualism: An Empirical Comparison of Two Theories of Psychopathology in Two Large Longitudinal Cohorts Supplemental material, sj-docx-22-cpx-10.1177_21677026231162814 for Common Cause Versus Dynamic Mutualism: An Empirical Comparison of Two Theories of Psychopathology in Two Large Longitudinal Cohorts by Michael E. Aristodemou, Rogier A. Kievit, Aja L. Murray, Manuel Eisner, Denis Ribeaud and Eiko I. Fried in Clinical Psychological Science

sj-docx-23-cpx-10.1177_21677026231162814 – Supplemental material for Common Cause Versus Dynamic Mutualism: An Empirical Comparison of Two Theories of Psychopathology in Two Large Longitudinal Cohorts Supplemental material, sj-docx-23-cpx-10.1177_21677026231162814 for Common Cause Versus Dynamic Mutualism: An Empirical Comparison of Two Theories of Psychopathology in Two Large Longitudinal Cohorts by Michael E. Aristodemou, Rogier A. Kievit, Aja L. Murray, Manuel Eisner, Denis Ribeaud and Eiko I. Fried in Clinical Psychological Science

sj-docx-24-cpx-10.1177_21677026231162814 – Supplemental material for Common Cause Versus Dynamic Mutualism: An Empirical Comparison of Two Theories of Psychopathology in Two Large Longitudinal Cohorts Supplemental material, sj-docx-24-cpx-10.1177_21677026231162814 for Common Cause Versus Dynamic Mutualism: An Empirical Comparison of Two Theories of Psychopathology in Two Large Longitudinal Cohorts by Michael E. Aristodemou, Rogier A. Kievit, Aja L. Murray, Manuel Eisner, Denis Ribeaud and Eiko I. Fried in Clinical Psychological Science

sj-docx-25-cpx-10.1177_21677026231162814 – Supplemental material for Common Cause Versus Dynamic Mutualism: An Empirical Comparison of Two Theories of Psychopathology in Two Large Longitudinal Cohorts Supplemental material, sj-docx-25-cpx-10.1177_21677026231162814 for Common Cause Versus Dynamic Mutualism: An Empirical Comparison of Two Theories of Psychopathology in Two Large Longitudinal Cohorts by Michael E. Aristodemou, Rogier A. Kievit, Aja L. Murray, Manuel Eisner, Denis Ribeaud and Eiko I. Fried in Clinical Psychological Science

sj-docx-3-cpx-10.1177_21677026231162814 – Supplemental material for Common Cause Versus Dynamic Mutualism: An Empirical Comparison of Two Theories of Psychopathology in Two Large Longitudinal Cohorts Supplemental material, sj-docx-3-cpx-10.1177_21677026231162814 for Common Cause Versus Dynamic Mutualism: An Empirical Comparison of Two Theories of Psychopathology in Two Large Longitudinal Cohorts by Michael E. Aristodemou, Rogier A. Kievit, Aja L. Murray, Manuel Eisner, Denis Ribeaud and Eiko I. Fried in Clinical Psychological Science

sj-docx-4-cpx-10.1177_21677026231162814 – Supplemental material for Common Cause Versus Dynamic Mutualism: An Empirical Comparison of Two Theories of Psychopathology in Two Large Longitudinal Cohorts Supplemental material, sj-docx-4-cpx-10.1177_21677026231162814 for Common Cause Versus Dynamic Mutualism: An Empirical Comparison of Two Theories of Psychopathology in Two Large Longitudinal Cohorts by Michael E. Aristodemou, Rogier A. Kievit, Aja L. Murray, Manuel Eisner, Denis Ribeaud and Eiko I. Fried in Clinical Psychological Science

sj-docx-5-cpx-10.1177_21677026231162814 – Supplemental material for Common Cause Versus Dynamic Mutualism: An Empirical Comparison of Two Theories of Psychopathology in Two Large Longitudinal Cohorts Supplemental material, sj-docx-5-cpx-10.1177_21677026231162814 for Common Cause Versus Dynamic Mutualism: An Empirical Comparison of Two Theories of Psychopathology in Two Large Longitudinal Cohorts by Michael E. Aristodemou, Rogier A. Kievit, Aja L. Murray, Manuel Eisner, Denis Ribeaud and Eiko I. Fried in Clinical Psychological Science

sj-docx-6-cpx-10.1177_21677026231162814 – Supplemental material for Common Cause Versus Dynamic Mutualism: An Empirical Comparison of Two Theories of Psychopathology in Two Large Longitudinal Cohorts Supplemental material, sj-docx-6-cpx-10.1177_21677026231162814 for Common Cause Versus Dynamic Mutualism: An Empirical Comparison of Two Theories of Psychopathology in Two Large Longitudinal Cohorts by Michael E. Aristodemou, Rogier A. Kievit, Aja L. Murray, Manuel Eisner, Denis Ribeaud and Eiko I. Fried in Clinical Psychological Science

sj-docx-7-cpx-10.1177_21677026231162814 – Supplemental material for Common Cause Versus Dynamic Mutualism: An Empirical Comparison of Two Theories of Psychopathology in Two Large Longitudinal Cohorts Supplemental material, sj-docx-7-cpx-10.1177_21677026231162814 for Common Cause Versus Dynamic Mutualism: An Empirical Comparison of Two Theories of Psychopathology in Two Large Longitudinal Cohorts by Michael E. Aristodemou, Rogier A. Kievit, Aja L. Murray, Manuel Eisner, Denis Ribeaud and Eiko I. Fried in Clinical Psychological Science

sj-docx-8-cpx-10.1177_21677026231162814 – Supplemental material for Common Cause Versus Dynamic Mutualism: An Empirical Comparison of Two Theories of Psychopathology in Two Large Longitudinal Cohorts Supplemental material, sj-docx-8-cpx-10.1177_21677026231162814 for Common Cause Versus Dynamic Mutualism: An Empirical Comparison of Two Theories of Psychopathology in Two Large Longitudinal Cohorts by Michael E. Aristodemou, Rogier A. Kievit, Aja L. Murray, Manuel Eisner, Denis Ribeaud and Eiko I. Fried in Clinical Psychological Science

sj-docx-9-cpx-10.1177_21677026231162814 – Supplemental material for Common Cause Versus Dynamic Mutualism: An Empirical Comparison of Two Theories of Psychopathology in Two Large Longitudinal Cohorts Supplemental material, sj-docx-9-cpx-10.1177_21677026231162814 for Common Cause Versus Dynamic Mutualism: An Empirical Comparison of Two Theories of Psychopathology in Two Large Longitudinal Cohorts by Michael E. Aristodemou, Rogier A. Kievit, Aja L. Murray, Manuel Eisner, Denis Ribeaud and Eiko I. Fried in Clinical Psychological Science

## Transparency

*Action Editor:* Jennifer L. Tackett

*Editor:* Jennifer L. Tackett

*Author Contributions*

**Michael E. Aristodemou:** Conceptualization; Formal analysis; Visualization; Writing – original draft; Writing – review & editing.

**Rogier A. Kievit:** Formal analysis; Resources; Supervision; Writing – review & editing.

**Aja L. Murray:** Conceptualization; Writing – review & editing.

**Manuel Eisner:** Investigation; Resources; Writing – review & editing.

**Denis Ribeaud:** Investigation; Resources; Writing – review & editing.

**Eiko I. Fried:** Conceptualization; Project administration; Resources; Supervision; Writing – review & editing.

**Open Practices:** This article has received the badges for Open Materials and Preregistration. More information about the Open Practices badges can be found at http://www.psychologicalscience.org/publications/badges.

## Notes

1. The notation “*b*” refers to the raw effect size; the Greek notation “β” refers to the standardized effect size.
