# Supplementary material for: Common Cause Versus Dynamic Mutualism: An Empirical Comparison of Two Theories of Psychopathology in Two Large Longitudinal Cohorts
Source: Clin Psychol Sci. 2023 May 25;12(3):380–402. doi: 10.1177/21677026231162814 (PMC11136614; doi:10.1177/21677026231162814)
Supplement: sj-docx-1-cpx-10.1177_21677026231162814 – Supplemental material for Common Cause Versus Dynamic Mutualism: An Empirical Comparison of Two Theories of Psychopathology in Two Large Longitudinal Cohorts [file sj-docx-1-cpx-10.1177_21677026231162814.docx]

| Table S1 *Partially invariant model comparison (z-proso)* | | | | | |
| --- | --- | --- | --- | --- | --- |
| Exploratory models | | | | | |
| Model | χ2 | df | RMSEA | CFI | SRMR |
| Common cause | < 0.001 | 13798 | 0.032 [0.032, 0.033] | 0.771 | 0.107 |
| Dynamic mutualism | < 0.001 | 13680 | 0.029 [0.029, 0.030] | 0.816 | 0.066 |
|  | | | | | |
