# Supplementary material for: Common Cause Versus Dynamic Mutualism: An Empirical Comparison of Two Theories of Psychopathology in Two Large Longitudinal Cohorts
Source: Clin Psychol Sci. 2023 May 25;12(3):380–402. doi: 10.1177/21677026231162814 (PMC11136614; doi:10.1177/21677026231162814)
Supplement: sj-docx-10-cpx-10.1177_21677026231162814 – Supplemental material for Common Cause Versus Dynamic Mutualism: An Empirical Comparison of Two Theories of Psychopathology in Two Large Longitudinal Cohorts [file sj-docx-10-cpx-10.1177_21677026231162814.docx]

| Table S10  EFA factor loadings for wave 2 | | | | | |
| --- | --- | --- | --- | --- | --- |
| Item | Description | Wave 2 | | | |
|  | | Factor 1 | Factor 2 | Factor 3 | Factor 4 |
| 606 | Force others | 0.63 |  |  |  |
| 608 | Violent attack | 0.79 |  |  |  |
| 629 | Aggressive when insulted | 0.79 |  |  |  |
| 613 | Hit, bite, kick others | 0.74 |  |  |  |
| 630 | Humiliate others | 0.59 |  |  |  |
| 618 | Aggressive when something taken | 0.67 |  |  |  |
| 619 | Threaten others | 0.57 |  |  |  |
| 622 | Engage in brawl | 0.70 |  |  |  |
| 615 | Yell at parent |  |  | 0.36 |  |
| 616 | Active exclusion | 0.38 |  |  |  |
| 633 | Told secrets | 0.36 |  |  |  |
| 621 | Throw thing at parent | 0.29 |  |  |  |
| 624 | Mad when not getting something | 0.41 |  |  |  |
| 603 | Aggressive when teased | 0.37 |  |  |  |
| 605 | Bad things behind back | 0.33 |  |  |  |
| 602 | Hit parent | 0.39 |  |  |  |
| 609 | Boss others | 0.46 |  |  |  |
| 610 | Lie to parent |  |  |  | 0.31 |
| 612 | Incite to dislike others | 0.42 |  |  |  |
| 652 | Cried |  |  | 0.70 |  |
| 653 | Fear |  |  | 0.64 |  |
| 654 | Unhappy |  |  | 0.78 |  |
| 655 | Felt alone |  |  | 0.78 |  |
| 657 | Sad without reason |  |  | 0.65 |  |
| 658 | Worried |  |  | 0.63 |  |
| 651 | Bored |  |  |  | 0.23 |
| 656 | Could not sleep |  |  | 0.39 |  |
| 659 | Self-injury |  |  | 0.42 |  |
| 627 | Restless |  |  |  | 0.64 |
| 628 | Difficulty concentrating |  |  |  | 0.69 |
| 631 | Inattentive |  |  |  | 0.63 |
| 632 | Hectic and fidgety |  |  |  | 0.66 |
| 601 | Help clear up |  | 0.41 |  |  |
| 604 | Understand others |  | 0.55 |  |  |
| 611 | Settle dispute |  | 0.56 |  |  |
| 614 | Feel sympathy |  | 0.74 |  |  |
| 617 | Help injured |  | 0.66 |  |  |
| 620 | Comfort |  | 0.78 |  |  |
| 625 | Sympathy for someone feeling bad |  | 0.80 |  |  |
| 626 | Sympathy for bullied |  | 0.64 |  |  |
| 623 | Listen to others |  | 0.48 |  |  |
| 607 | Share with others |  | 0.46 |  |  |

Narrative summary of wave 2: All items loaded highest onto their CFA factors—apart from the (1) externalizing items: 610: “lie to parent” and 615: “yell at parent” which loaded onto the internalizing and ADHD factors, respectively. (2) The internalizing item 651 “bored” loaded onto the ADHD factor.
