# Supplementary material for: Common Cause Versus Dynamic Mutualism: An Empirical Comparison of Two Theories of Psychopathology in Two Large Longitudinal Cohorts
Source: Clin Psychol Sci. 2023 May 25;12(3):380–402. doi: 10.1177/21677026231162814 (PMC11136614; doi:10.1177/21677026231162814)
Supplement: sj-docx-12-cpx-10.1177_21677026231162814 – Supplemental material for Common Cause Versus Dynamic Mutualism: An Empirical Comparison of Two Theories of Psychopathology in Two Large Longitudinal Cohorts [file sj-docx-12-cpx-10.1177_21677026231162814.docx]

| Table S12  EFA factor loadings for wave 4 | | | | | |
| --- | --- | --- | --- | --- | --- |
| Item | Description | Wave 4 | | | |
|  | | Factor 1 | Factor 2 | Factor 3 | Factor 4 |
| 606 | Force others |  |  |  | 0.49 |
| 608 | Violent attack |  |  | 0.84 |  |
| 629 | Aggressive when insulted |  |  | 0.41 |  |
| 613 | Hit, bite, kick others |  |  | 0.78 |  |
| 630 | Humiliate others |  |  |  | 0.42 |
| 618 | Aggressive when something taken |  |  | 0.55 |  |
| 619 | Threaten others |  |  | 0.40 |  |
| 622 | Engage in brawl |  |  | 0.77 |  |
| 615 | Yell at parent | 0.39 |  |  |  |
| 616 | Active exclusion |  |  |  | 0.66 |
| 633 | Told secrets |  |  |  | 0.61 |
| 621 | Throw thing at parent |  |  | 0.25 | 0.25 |
| 624 | Mad when not getting something |  |  |  | 0.32 |
| 603 | Aggressive when teased | 0.37 |  |  |  |
| 605 | Bad things behind back |  |  |  | 0.62 |
| 602 | Hit parent |  |  | 0.23 | 0.23 |
| 609 | Boss others |  |  |  | 0.35 |
| 610 | Lie to parent | 0.40 |  |  |  |
| 612 | Incite to dislike others |  |  |  | 0.69 |
| 652 | Cried | 0.65 |  |  |  |
| 653 | Fear | 0.70 |  |  |  |
| 654 | Unhappy | 0.74 |  |  |  |
| 655 | Felt alone | 0.73 |  |  |  |
| 657 | Sad without reason | 0.72 |  |  |  |
| 658 | Worried | 0.73 |  |  |  |
| 651 | Bored | 0.42 |  |  |  |
| 656 | Could not sleep | 0.59 |  |  |  |
| 659 | Self-injury | 0.40 |  |  |  |
| 627 | Restless | 0.42 |  |  |  |
| 628 | Difficulty concentrating | 0.55 |  |  |  |
| 631 | Inattentive | 0.55 |  |  |  |
| 632 | Hectic and fidgety | 0.50 |  |  |  |
| 601 | Help clear up |  | 0.41 |  |  |
| 604 | Understand others |  | 0.54 |  |  |
| 611 | Settle dispute |  | 0.57 |  |  |
| 614 | Feel sympathy |  | 0.72 |  |  |
| 617 | Help injured |  | 0.71 |  |  |
| 620 | Comfort |  | 0.77 |  |  |
| 625 | Sympathy for someone feeling bad |  | 0.75 |  |  |
| 626 | Sympathy for bullied |  | 0.56 |  |  |
| 623 | Listen to others |  | 0.43 |  |  |
| 607 | Share with others |  | 0.49 |  |  |

Narrative summary of wave 4: (a) Internalizing and ADHD items loaded highest on the same factor, along with 3 externalizing items: 615 “yell at parent”, 610 “lie to parent”, 603 “aggressive when teased”. (b) The rest of the externalizing items were spread across two factors. (c) All pro-sociality items loaded on the same factor.
