# Supplementary material for: Common Cause Versus Dynamic Mutualism: An Empirical Comparison of Two Theories of Psychopathology in Two Large Longitudinal Cohorts
Source: Clin Psychol Sci. 2023 May 25;12(3):380–402. doi: 10.1177/21677026231162814 (PMC11136614; doi:10.1177/21677026231162814)
Supplement: sj-docx-13-cpx-10.1177_21677026231162814 – Supplemental material for Common Cause Versus Dynamic Mutualism: An Empirical Comparison of Two Theories of Psychopathology in Two Large Longitudinal Cohorts [file sj-docx-13-cpx-10.1177_21677026231162814.docx]

| Table S13  *Standardized Factor loadings for four first-order factors* | | |  |  |  |  |
| --- | --- | --- | --- | --- | --- | --- |
| Items | Abbreviated content | Wave 1 | | Wave 2 | Wave 3 | Wave 4 |
| Prosociality |  | | | | | |
| K_614 | Feel sympathy | 0.780 | | 0.788 | 0.801 | 0.775 |
| K_604 | Understand feelings | 0.528 | | 0.546 | 0.575 | 0.505 |
| K_607 | Share with others | 0.433 | | 0.424 | 0.432 | 0.403 |
| K_611 | Settle dispute | 0.512 | | 0.479 | 0.506 | 0.455 |
| K_620 | Try to comfort | 0.402 | | 0.365 | 0.376 | 0.295 |
| K_617 | Try to help injured | 0.678 | | 0.633 | 0.627 | 0.690 |
| K_601 | Help clear up | 0.792 | | 0.772 | 0.776 | 0.773 |
| K_625 | Sympathy for feel bad | 0.840 | | 0.833 | 0.843 | 0.818 |
| K_623 | Listen to other opinion | 0.491 | | 0.478 | 0.509 | 0.463 |
| K_626 | Sympathy for bullied | 0.701 | | 0.687 | 0.711 | 0.603 |
| Externalizing |  | | | | | |
| K_618 | Aggressive if something taken | 0.688 | | 0.677 | 0.685 | 0.524 |
| K_603 | Aggressive when teased | 0.446 | | 0.421 | 0.360 | 0.317 |
| K_605 | Bad things behind back | 0.444 | | 0.431 | 0.392 | 0.370 |
| K_602 | Hit parent | 0.676 | | 0.640 | 0.604 | 0.590 |
| K_608 | Violent attack | 0.756 | | 0.750 | 0.770 | 0.742 |
| K_609 | Boss others around | 0.507 | | 0.474 | 0.381 | 0.299 |
| K_629 | Aggressive when insulted | 0.741 | | 0.749 | 0.749 | 0.405 |
| K_610 | Lie to parent | 0.360 | | 0.299 | 0.263 | 0.235 |
| K_612 | Incite other to dislike | 0.524 | | 0.476 | 0.453 | 0.437 |
| K_613 | Hit, bite, kick others | 0.725 | | 0.733 | 0.726 | 0.759 |
| K_630 | Humiliate others | 0.671 | | 0.653 | 0.653 | 0.663 |
| K_615 | Yell at parent | 0.350 | | 0.305 | 0.278 | 0.249 |
| K_616 | Active exclusion | 0.472 | | 0.461 | 0.452 | 0.514 |
| K_633 | Told secrets when mad | 0.442 | | 0.446 | 0.431 | 0.415 |
| K_606 | Scare to force others | 0.371 | | 0.350 | 0.407 | 0.396 |
| K_619 | Threat others to get something | 0.596 | | 0.574 | 0.580 | 0.595 |
| K_621 | Throw things at parent | 0.402 | | 0.365 | 0.344 | 0.380 |
| K_622 | Engage in brawl | 0.666 | | 0.644 | 0.665 | 0.675 |
| K_624 | Mad not getting something | 0.513 | | 0.475 | 0.446 | 0.405 |
| Internalizing |  | | | | | |
| K_657 | Sad without reason | 0.657 | | 0.656 | 0.676 | 0.708 |
| K_652 | Cried | 0.644 | | 0.661 | 0.662 | 0.664 |
| K_653 | Fear | 0.636 | | 0.655 | 0.639 | 0.650 |
| K_654 | Unhappy | 0.749 | | 0.773 | 0.786 | 0.790 |
| K_651 | Bored | 0.743 | | 0.761 | 0.755 | 0.763 |
| K_656 | Could not fall asleep | 0.481 | | 0.512 | 0.534 | 0.547 |
| K_655 | Felt alone | 0.272 | | 0.307 | 0.311 | 0.310 |
| K_658 | Worried | 0.662 | | 0.699 | 0.723 | 0.761 |
| K_659 | Self-injury | 0.309 | | 0.352 | 0.376 | 0.453 |
| ADHD |  | | | | | |
| K_627 | Restless | 0.640 | | 0.687 | 0.717 | 0.698 |
| K_628 | Difficulties to concentrate | 0.591 | | 0.665 | 0.658 | 0.654 |
| K_631 | Inattentive | 0.525 | | 0.567 | 0.598 | 0.651 |
| K_632 | Hectic and fidgety | 0.682 | | 0.730 | 0.751 | 0.760 |
| Note: Cross-loadings in confirmatory factor model were constrained to zero. | | | | | | |
