# Supplementary material for: Common Cause Versus Dynamic Mutualism: An Empirical Comparison of Two Theories of Psychopathology in Two Large Longitudinal Cohorts
Source: Clin Psychol Sci. 2023 May 25;12(3):380–402. doi: 10.1177/21677026231162814 (PMC11136614; doi:10.1177/21677026231162814)
Supplement: sj-docx-14-cpx-10.1177_21677026231162814 – Supplemental material for Common Cause Versus Dynamic Mutualism: An Empirical Comparison of Two Theories of Psychopathology in Two Large Longitudinal Cohorts [file sj-docx-14-cpx-10.1177_21677026231162814.docx]

| Table S14  *Partially invariant model raw estimates of item intercepts* | | |  |  |  |  |
| --- | --- | --- | --- | --- | --- | --- |
| Items | Abbreviated content | Wave 1 | | Wave 2 | Wave 3 | Wave 4 |
| Prosociality |  | | | | | |
| K_614 | Feel sympathy | 0.000 | | 0.000 | 0.000 | 0.000 |
| K_604 | Understand feelings | 0.606 | | 0.606 | 0.606 | 0.606 |
| K_607 | Share with others | 1.134 | | 1.134 | 1.134 | 1.134 |
| K_611 | Settle dispute | 1.328 | | 1.328 | 1.328 | 1.328 |
| K_620 | Try to comfort | -0.101 | | -0.101 | -0.101 | -0.101 |
| K_617 | Try to help injured | 0.371 | | 0.442 | 0.536 | 0.212 |
| K_601 | Help clear up | 1.749 | | 1.742 | 1.534 | 1.910 |
| K_625 | Sympathy for feel bad | -0.208 | | -0.208 | -0.208 | -0.208 |
| K_623 | Listen to other opinion | 1.219 | | 1.153 | 0.918 | 0.832 |
| K_626 | Sympathy for bullied | 0.390 | | 0.390 | 0.390 | 0.390 |
| Externalizing |  | | | | | |
| K_618 | Aggressive if something taken | 0.000 | | 0.000 | 0.000 | 0.000 |
| K_603 | Aggressive when teased | 1.623 | | 1.623 | 1.623 | 1.623 |
| K_605 | Bad things behind back | 0.717 | | 0.717 | 0.717 | 0.717 |
| K_602 | Hit parent | 0.656 | | 0.656 | 0.656 | 0.656 |
| K_608 | Violent attack | -0.168 | | -0.168 | -0.168 | -0.168 |
| K_609 | Boss others around | 0.413 | | 0.531 | 0.703 | 0.653 |
| K_629 | Aggressive when insulted | -0.233 | | -0.285 | -0.309 | 0.422 |
| K_610 | Lie to parent | 1.311 | | 1.609 | 1.688 | 1.410 |
| K_612 | Incite other to dislike | 0.460 | | 0.460 | 0.460 | 0.460 |
| K_613 | Hit, bite, kick others | -0.178 | | -0.178 | -0.178 | -0.178 |
| K_630 | Humiliate others | 0.204 | | 0.181 | 0.065 | -0.045 |
| K_615 | Yell at parent | 1.276 | | 1.276 | 1.276 | 1.276 |
| K_616 | Active exclusion | 0.767 | | 0.645 | 0.575 | 0.352 |
| K_633 | Told secrets when mad | 0.511 | | 0.511 | 0.511 | 0.511 |
| K_606 | Scare to force others | -0.068 | | 0.020 | 0.092 | 0.033 |
| K_619 | Threat others to get something | 0.325 | | 0.325 | 0.325 | 0.325 |
| K_621 | Throw things at parent | 0.642 | | 0.642 | 0.642 | 0.642 |
| K_622 | Engage in brawl | -0.066 | | -0.066 | -0.066 | -0.066 |
| K_624 | Mad not getting something | 0.537 | | 0.537 | 0.537 | 0.537 |
| Internalizing |  | | | | | |
| K_657 | Sad without reason | 0.000 | | 0.000 | 0.000 | 0.000 |
| K_652 | Cried | 0.288 | | 0.288 | 0.288 | 0.288 |
| K_653 | Fear | 0.116 | | 0.116 | 0.116 | 0.116 |
| K_654 | Unhappy | 0.230 | | 0.230 | 0.230 | 0.230 |
| K_651 | Bored | 2.188 | | 2.055 | 1.888 | 1.846 |
| K_656 | Could not fall asleep | 0.870 | | 0.870 | 0.870 | 0.870 |
| K_655 | Felt alone | -0.152 | | -0.152 | -0.152 | -0.152 |
| K_658 | Worried | 0.361 | | 0.552 | 0.607 | 0.668 |
| K_659 | Self-injury | 0.604 | | 0.604 | 0.604 | 0.604 |
| ADHD |  | | | | | |
| K_627 | Restless | 0.000 | | 0.000 | 0.000 | 0.000 |
| K_628 | Difficulties to concentrate | 0.149 | | 0.147 | 0.270 | 0.432 |
| K_631 | Inattentive | 0.619 | | 0.619 | 0.619 | 0.619 |
| K_632 | Hectic and fidgety | -0.411 | | -0.411 | -0.411 | -0.411 |
| *Note: Item intercepts were sequentially freed based on modification indices until measurement partial measurement invariance was achieved. We highlighted the non-invariant intercepts within each factor using a different color. | | | | | | |
