# Supplementary material for: Common Cause Versus Dynamic Mutualism: An Empirical Comparison of Two Theories of Psychopathology in Two Large Longitudinal Cohorts
Source: Clin Psychol Sci. 2023 May 25;12(3):380–402. doi: 10.1177/21677026231162814 (PMC11136614; doi:10.1177/21677026231162814)
Supplement: sj-docx-15-cpx-10.1177_21677026231162814 – Supplemental material for Common Cause Versus Dynamic Mutualism: An Empirical Comparison of Two Theories of Psychopathology in Two Large Longitudinal Cohorts [file sj-docx-15-cpx-10.1177_21677026231162814.docx]

| Table S15  *Self-feedback parameters for common cause model (z-proso)* | | | | | | | |
| --- | --- | --- | --- | --- | --- | --- | --- |
| Regressions | Estimate | Std.Err | z-value | P(>\|z\|) | ci.lower | ci.upper | *β* |
| Δpfactor at T2 regressed on ~ |  |  |  |  |  |  |  |
| pfactor T1 | -0.008 | 0.069 | -0.114 | 0.909 | -0.144 | 0.128 | -0.013 |
| Δpfactor at T3 regressed on ~ |  |  |  |  |  |  |  |
| Dpfactor T2 | -0.067 | 0.038 | -1.730 | 0.084 | -0.142 | 0.009 | -0.119 |
| Δpfactor at T4 regressed on ~ |  |  |  |  |  |  |  |
| pfactor T3 | -0.157 | 0.036 | -4.393 | 0.000 | -0.228 | -0.087 | -0.266 |

*Note: Δ represents the latent variable that captures change between time points, e.g. Δpfactor at T2 represents the change between the p-factor scores at T1 and the p-factor scores at T2.
