# Supplementary material for: Common Cause Versus Dynamic Mutualism: An Empirical Comparison of Two Theories of Psychopathology in Two Large Longitudinal Cohorts
Source: Clin Psychol Sci. 2023 May 25;12(3):380–402. doi: 10.1177/21677026231162814 (PMC11136614; doi:10.1177/21677026231162814)
Supplement: sj-docx-16-cpx-10.1177_21677026231162814 – Supplemental material for Common Cause Versus Dynamic Mutualism: An Empirical Comparison of Two Theories of Psychopathology in Two Large Longitudinal Cohorts [file sj-docx-16-cpx-10.1177_21677026231162814.docx]

| Table S16  *Change score variances for common cause model (z-proso)* | | | | | | | |
| --- | --- | --- | --- | --- | --- | --- | --- |
| Change scores | Estimate | Std.Err | z-value | P(>\|z\|) | ci.lower | ci.upper | *β* |
| Δpfactor at T2 | 0.052 | 0.013 | 4.168 | 0.000 | 0.028 | 0.077 | 1.000 |
| Δpfactor at T3 | 0.061 | 0.011 | 5.628 | 0.000 | 0.039 | 0.082 | 0.986 |
| Δpfactor at T4 | 0.076 | 0.014 | 5.358 | 0.000 | 0.048 | 0.103 | 0.929 |

*Note: Δ represents the latent variable that captures change between time points, e.g. Δpfactor at T2 represents the change between the p-factor scores at T1 and the p-factor scores at T2.
