# Supplementary material for: Common Cause Versus Dynamic Mutualism: An Empirical Comparison of Two Theories of Psychopathology in Two Large Longitudinal Cohorts
Source: Clin Psychol Sci. 2023 May 25;12(3):380–402. doi: 10.1177/21677026231162814 (PMC11136614; doi:10.1177/21677026231162814)
Supplement: sj-docx-17-cpx-10.1177_21677026231162814 – Supplemental material for Common Cause Versus Dynamic Mutualism: An Empirical Comparison of Two Theories of Psychopathology in Two Large Longitudinal Cohorts [file sj-docx-17-cpx-10.1177_21677026231162814.docx]

| Table S17  *Model comparison fit statistics for z-proso models with gender as a covariate* | | | | | |  |  |
| --- | --- | --- | --- | --- | --- | --- | --- |
| Model | χ2 | Df | RMSEA | CFI | SRMR | AIC | BIC |
| Common cause | 33268.304 | 14002 | 0.033 [0.032, 0.033] | 0.731 | 0.097 | 551994.023 | 554814.234 |
| Mutualism | 29458.188 | 13880 | 0.029 [0.029, 0.030] | 0.783 | 0.062 | 547761.664 | 551228.615 |
