# Supplementary material for: Common Cause Versus Dynamic Mutualism: An Empirical Comparison of Two Theories of Psychopathology in Two Large Longitudinal Cohorts
Source: Clin Psychol Sci. 2023 May 25;12(3):380–402. doi: 10.1177/21677026231162814 (PMC11136614; doi:10.1177/21677026231162814)
Supplement: sj-docx-18-cpx-10.1177_21677026231162814 – Supplemental material for Common Cause Versus Dynamic Mutualism: An Empirical Comparison of Two Theories of Psychopathology in Two Large Longitudinal Cohorts [file sj-docx-18-cpx-10.1177_21677026231162814.docx]

| Table S18  *Regression parameters for dynamic mutualism model with gender as covariate at T1 (z-proso)* | | | | | | | | |
| --- | --- | --- | --- | --- | --- | --- | --- | --- |
| Regressions | Estimate | | Std.Err | z-value | P(>\|z\|) | ci.lower | ci.upper | *β*** |
| ***Δinternalizing at T2 ~ regressed on** |  | |  |  |  |  |  |  |
| Internalizing factor T1 | -0.364 | | 0.048 | -7.525 | 0.000 | -0.459 | -0.269 | -0.374 |
| Externalizing factor T1 | -0.112 | | 0.066 | -1.698 | 0.089 | -0.241 | 0.017 | -0.098 |
| Prosociality factor T1 | 0.044 | | 0.044 | 1.018 | 0.309 | -0.041 | 0.130 | 0.050 |
| ADHD factor T1 | 0.048 | | 0.074 | 0.651 | 0.515 | -0.097 | 0.193 | 0.045 |
| **Δinternalizing at T3 ~ regressed on** | |  |  |  |  |  |  |  |
| Internalizing factor T2 | -0.208 | | 0.086 | -2.411 | 0.016 | -0.377 | -0.039 | -0.240 |
| Externalizing factor T2 | -0.065 | | 0.118 | -0.552 | 0.581 | -0.296 | 0.166 | -0.051 |
| Prosociality factor T2 | 0.095 | | 0.102 | 0.933 | 0.351 | -0.105 | 0.295 | 0.101 |
| ADHD factor T2 | 0.127 | | 0.112 | 1.139 | 0.255 | -0.092 | 0.346 | 0.122 |
| **Δinternalizing at T4 ~ regressed on** |  | |  |  |  |  |  |  |
| Internalizing factor T3 | -0.207 | | 0.116 | -1.781 | 0.075 | -0.436 | 0.021 | -0.239 |
| Externalizing factor T3 | -0.064 | | 0.161 | -0.397 | 0.692 | -0.380 | 0.252 | -0.042 |
| Prosociality factor T3 | -0.143 | | 0.116 | -1.234 | 0.217 | -0.369 | 0.084 | 0.147 |
| ADHD factor T3 | 0.084 | | 0.139 | 0.602 | 0.547 | -0.189 | 0.357 | 0.084 |
| **Δexternalizing at T2 ~ regressed on** |  | |  |  |  |  |  |  |
| Internalizing factor T1 | 0.016 | | 0.032 | 0.500 | 0.617 | -0.047 | 0.079 | 0.021 |
| Externalizing factor T1 | -0.490 | | 0.062 | -7.896 | 0.000 | -0.612 | -0.369 | -0.556 |
| Prosociality factor T1 | -0.094 | | 0.040 | -2.334 | 0.020 | -0.173 | -0.015 | -0.137 |
| ADHD factor T1 | -0.079 | | 0.044 | -1.782 | 0.075 | -0.165 | 0.008 | -0.096 |
| **Δexternalizing at T3 ~ regressed on** |  | |  |  |  |  |  |  |
| Internalizing factor T2 | 0.023 | | 0.050 | 0.458 | 0.647 | -0.075 | 0.120 | 0.042 |
| Externalizing factor T2 | -0.272 | | 0.073 | -3.737 | 0.000 | -0.414 | -0.129 | -0.338 |
| Prosociality factor T2 | -0.027 | | 0.056 | -0.475 | 0.635 | -0.136 | 0.083 | -0.045 |
| ADHD factor T2 | -0.020 | | 0.059 | -0.333 | 0.739 | -0.136 | 0.096 | -0.030 |
| **Δexternalizing at T4 ~ regressed on** |  | |  |  |  |  |  |  |
| Internalizing factor T3 | 0.001 | | 0.047 | 0.021 | 0.983 | -0.091 | 0.093 | 0.002 |
| Externalizing factor T3 | -0.433 | | 0.077 | -5.596 | 0.000 | -0.584 | -0.281 | -0.564 |
| Prosociality factor T3 | 0.064 | | 0.050 | 1.296 | 0.195 | -0.033 | 0.162 | -0.094 |
| ADHD factor T3 | -0.038 | | 0.047 | -0.799 | 0.424 | -0.130 | 0.055 | 0.042 |
| **Δprosociality at T2 ~ regressed on** |  | |  |  |  |  |  |  |
| Internalizing factor T1 | 0.099 | | 0.035 | 2.789 | 0.005 | 0.029 | 0.168 | 0.101 |
| Externalizing factor T1 | -0.124 | | 0.053 | -2.327 | 0.020 | -0.228 | -0.019 | -0.108 |
| Prosociality factor T1 | -0.497 | | 0.036 | -13.776 | 0.000 | -0.567 | -0.426 | -0.557 |
| ADHD factor T1 | -0.037 | | 0.053 | -0.700 | 0.484 | -0.141 | 0.067 | -0.035 |
| **Δprosociality at T3 ~ regressed on** |  | |  |  |  |  |  |  |
| Internalizing factor T2 | -0.026 | | 0.072 | -0.360 | 0.719 | -0.168 | 0.116 | -0.034 |
| Externalizing factor T2 | 0.033 | | 0.099 | 0.334 | 0.739 | -0.160 | 0.226 | 0.029 |
| Prosociality factor T2 | -0.164 | | 0.080 | -2.038 | 0.042 | -0.322 | -0.006 | -0.196 |
| ADHD factor T2 | 0.042 | | 0.098 | 0.427 | 0.669 | -0.151 | 0.235 | 0.045 |
| **Δprosociality at T4 ~ regressed on** |  | |  |  |  |  |  |  |
| Internalizing factor T3 | 0.170 | | 0.092 | 1.863 | 0.062 | -0.009 | 0.350 | 0.255 |
| Externalizing factor T3 | -0.121 | | 0.140 | -0.865 | 0.387 | -0.396 | 0.154 | -0.091 |
| Prosociality factor T3 | -0.467 | | 0.097 | -4.796 | 0.000 | -0.657 | -0.276 | -0.550 |
| ADHD factor T3 | -0.005 | | 0.118 | -0.043 | 0.966 | -0.236 | 0.255 | -0.006 |
| **ΔADHD at T2 ~ regressed on** |  | |  |  |  |  |  |  |
| Internalizing factor T1 | 0.122 | | 0.042 | 2.922 | 0.003 | 0.040 | 0.203 | 0.136 |
| Externalizing factor T1 | -0.125 | | 0.063 | -1.976 | 0.048 | -0.250 | -0.001 | -0.120 |
| Prosociality factor T1 | -0.051 | | 0.040 | -1.265 | 0.206 | -0.130 | 0.028 | -0.063 |
| ADHD factor T1 | -0.451 | | 0.058 | -7.785 | 0.000 | -0.565 | -0.338 | -0.464 |
| **ΔADHD at T3 ~ regressed on** |  | |  |  |  |  |  |  |
| Internalizing factor T2 | 0.055 | | 0.079 | 0.705 | 0.481 | -0.099 | 0.210 | 0.074 |
| Externalizing factor T2 | 0.070 | | 0.110 | 0.635 | 0.526 | -0.145 | 0.284 | 0.063 |
| Prosociality factor T2 | -0.018 | | 0.084 | -0.217 | 0.828 | -0.183 | 0.147 | -0.022 |
| ADHD factor T1 | -0.168 | | 0.106 | 1.585 | 0.113 | -0.375 | 0.040 | -0.186 |
| **ΔADHD at T4 ~ regressed on** |  | |  |  |  |  |  |  |
| Internalizing factor T3 | 0.155 | | 0.103 | 1.499 | 0.134 | -0.048 | 0.357 | 0.214 |
| Externalizing factor T3 | -0.033 | | 0.146 | -0.227 | 0.820 | -0.319 | 0.253 | -0.026 |
| Prosociality factor T3 | 0.022 | | 0.111 | 0.201 | 0.841 | -0.195 | 0.239 | 0.027 |
| ADHD factor T3 | -0.320 | | 0.126 | -2.551 | 0.011 | -0.566 | -0.074 | -0.384 |
| *Note: Δ indicates a change score, for instance ΔADHD at T2 indicates the latent variable that captures the change between the ADHD factor at T1 and the ADHD factor at T2.  **Note: *β* refers to the standardized regression coefficient. | | | | | | | | |

| Table S18B  Gender as a covariate of psychopathology dimensions at T1 (z-proso) | | | | | | | |
| --- | --- | --- | --- | --- | --- | --- | --- |
| Regressions | Estimate | Std. Error | z-value | P(>\|z\|) | CI lower | CI upper | β |
| **(Psychopatholgy T1) ~ Gender** | | | | | | | |
| Internalizing T1 | 0.498 | 0.046 | 10.782 | 0.000 | 0.408 | 0.589 | 0.322 |
| Externalizing T1 | -0.390 | 0.041 | -9.554 | 0.000 | -0.470 | -0.310 | -0.304 |
| Prosociality T1 | 0.691 | 0.045 | 15.212 | 0.000 | 0.602 | 0.780 | 0.418 |
| ADHD T1 | 0.063 | 0.056 | 1.122 | 0.262 | -0.047 | 0.174 | 0.046 |
|  |  |  |  |  |  |  |  |
