# Supplementary material for: Common Cause Versus Dynamic Mutualism: An Empirical Comparison of Two Theories of Psychopathology in Two Large Longitudinal Cohorts
Source: Clin Psychol Sci. 2023 May 25;12(3):380–402. doi: 10.1177/21677026231162814 (PMC11136614; doi:10.1177/21677026231162814)
Supplement: sj-docx-19-cpx-10.1177_21677026231162814 – Supplemental material for Common Cause Versus Dynamic Mutualism: An Empirical Comparison of Two Theories of Psychopathology in Two Large Longitudinal Cohorts [file sj-docx-19-cpx-10.1177_21677026231162814.docx]

| Table S19A  *Self-feedback parameters for common cause model (z-proso)* | | | | | | | |
| --- | --- | --- | --- | --- | --- | --- | --- |
| Regressions | Estimate | Std.Err | z-value | P(>\|z\|) | ci.lower | ci.upper | *β* |
| Δpfactor at T2 regressed on ~ |  |  |  |  |  |  |  |
| pfactor T1 | 0.166 | 0.093 | 1.792 | 0.073 | -0.016 | 0.347 | 0.341 |
| Δpfactor at T3 regressed on ~ |  |  |  |  |  |  |  |
| Dpfactor T2 | -0.064 | 0.053 | -1.216 | 0.224 | -0.168 | 0.039 | -0.144 |
| Δpfactor at T4 regressed on ~ |  |  |  |  |  |  |  |
| Dpfactor T3 | -0.173 | 0.035 | -4.967 | 0.000 | -0.242 | -0.105 | -0.299 |

| Table S19B  *Gender as covariate of pfactor at T1 (z-proso)* | | | | | | | |
| --- | --- | --- | --- | --- | --- | --- | --- |
| Regressions | Estimate | Std. Error | z-value | P(>\|z\|) | CI lower | CI upper | Beta |
| pfactor T1 ~ Gender | 0.314 | 0.025 | 12.329 | 0.000 | 0.264 | 0.364 | 0.502 |
|  |  |  |  |  |  |  |  |
