# Supplementary material for: Common Cause Versus Dynamic Mutualism: An Empirical Comparison of Two Theories of Psychopathology in Two Large Longitudinal Cohorts
Source: Clin Psychol Sci. 2023 May 25;12(3):380–402. doi: 10.1177/21677026231162814 (PMC11136614; doi:10.1177/21677026231162814)
Supplement: sj-docx-2-cpx-10.1177_21677026231162814 – Supplemental material for Common Cause Versus Dynamic Mutualism: An Empirical Comparison of Two Theories of Psychopathology in Two Large Longitudinal Cohorts [file sj-docx-2-cpx-10.1177_21677026231162814.docx]

| Table S2  *Regression parameters for dynamic mutualism model (z-proso)* | | | | | | | | |
| --- | --- | --- | --- | --- | --- | --- | --- | --- |
| Regressions | Estimate | | Std.Err | z-value | P(>\|z\|) | ci.lower | ci.upper | *β*** |
| ***Δinternalizing at T2 ~ regressed on** |  | |  |  |  |  |  |  |
| Internalizing factor T1 | -0.385 | | 0.044 | -8.822 | 0.000 | -0.470 | -0.299 | -0.400 |
| Externalizing factor T1 | -0.146 | | 0.050 | -2.916 | 0.004 | -0.244 | -0.048 | -0.125 |
| Prosociality factor T1 | -0.032 | | 0.031 | -1.044 | 0.297 | -0.093 | 0.028 | -0.037 |
| ADHD factor T1 | 0.094 | | 0.049 | 1.927 | 0.054 | -0.002 | 0.189 | 0.092 |
| **Δinternalizing at T3 ~ regressed on** | |  |  |  |  |  |  |  |
| Internalizing factor T2 | -0.168 | | 0.077 | -2.185 | 0.029 | -0.318 | -0.017 | -0.197 |
| Externalizing factor T2 | 0.011 | | 0.087 | 0.122 | 0.903 | -0.160 | 0.181 | 0.009 |
| Prosociality factor T2 | -0.093 | | 0.064 | -1.446 | 0.148 | -0.219 | 0.033 | -0.103 |
| ADHD factor T2 | 0.020 | | 0.082 | 0.243 | 0.808 | -0.142 | 0.182 | 0.021 |
| **Δinternalizing at T4 ~ regressed on** |  | |  |  |  |  |  |  |
| Internalizing factor T3 | -0.188 | | 0.109 | -1.727 | 0.084 | -0.401 | 0.025 | -0.214 |
| Externalizing factor T3 | -0.136 | | 0.143 | -0.955 | 0.340 | -0.416 | 0.143 | -0.089 |
| Prosociality factor T3 | 0.205 | | 0.101 | 2.039 | 0.041 | 0.008 | 0.402 | 0.213 |
| ADHD factor T3 | 0.079 | | 0.111 | 0.714 | 0.475 | -0.139 | 0.298 | 0.083 |
| **Δexternalizing at T2 ~ regressed on** |  | |  |  |  |  |  |  |
| Externalizing factor T1 | -0.464 | | 0.042 | 11.099 | 0.000 | -0.546 | -0.382 | -0.539 |
| Internalizing factor T1 | -0.002 | | 0.027 | -0.079 | 0.937 | -0.054 | 0.050 | -0.003 |
| Prosociality factor T1 | 0.059 | | 0.022 | 2.668 | 0.008 | 0.016 | 0.102 | 0.092 |
| ADHD factor T1 | -0.036 | | 0.032 | -1.129 | 0.259 | -0.100 | 0.027 | -0.049 |
| **Δexternalizing at T3 ~ regressed on** |  | |  |  |  |  |  |  |
| Externalizing factor T2 | -0.273 | | 0.071 | -3.871 | 0.000 | -0.411 | -0.135 | -0.341 |
| Internalizing factor T2 | 0.023 | | 0.047 | 0.483 | 0.629 | -0.070 | 0.115 | 0.042 |
| Prosociality factor T2 | 0.049 | | 0.044 | 1.107 | 0.268 | -0.038 | 0.135 | 0.084 |
| ADHD factor T2 | -0.008 | | 0.047 | -0.161 | 0.872 | -0.100 | 0.085 | -0.013 |
| **Δexternalizing at T4 ~ regressed on** |  | |  |  |  |  |  |  |
| Externalizing factor T3 | -0.433 | | 0.072 | -5.992 | 0.000 | -0.575 | -0.292 | -0.550 |
| Internalizing factor T3 | 0.021 | | 0.045 | 0.481 | 0.631 | -0.066 | 0.109 | 0.047 |
| Prosociality factor T3 | 0.064 | | 0.050 | 1.296 | 0.195 | -0.033 | 0.162 | 0.130 |
| ADHD factor T3 | -0.038 | | 0.047 | -0.799 | 0.424 | -0.130 | 0.055 | -0.077 |
| **Δprosociality at T2 ~ regressed on** |  | |  |  |  |  |  |  |
| Prosociality factor T1 | -0.454 | | 0.029 | 15.702 | 0.000 | -0.511 | -0.397 | -0.530 |
| Internalizing factor T1 | -0.064 | | 0.032 | -1.995 | 0.046 | -0.127 | -0.001 | -0.067 |
| Externalizing factor T1 | 0.041 | | 0.047 | 0.862 | 0.389 | -0.052 | 0.134 | 0.035 |
| ADHD factor T1 | 0.023 | | 0.038 | 0.621 | 0.534 | -0.051 | 0.097 | 0.023 |
| **Δprosociality at T3 ~ regressed on** |  | |  |  |  |  |  |  |
| Prosociality factor T2 | -0.208 | | 0.065 | -3.198 | 0.001 | -0.336 | -0.081 | -0.248 |
| Internalizing factor T2 | -0.030 | | 0.068 | -0.447 | 0.655 | -0.163 | 0.103 | -0.038 |
| Externalizing factor T2 | -0.030 | | 0.085 | -0.348 | 0.728 | -0.197 | 0.138 | -0.026 |
| ADHD factor T2 | 0.024 | | 0.072 | 0.329 | 0.742 | -0.118 | 0.165 | 0.027 |
| **Δprosociality at T4 ~ regressed on** |  | |  |  |  |  |  |  |
| Prosociality factor T3 | -0.446 | | 0.085 | -5.247 | 0.000 | -0.613 | -0.280 | -0.525 |
| Internalizing factor T3 | -0.162 | | 0.083 | -1.953 | 0.051 | -0.324 | 0.001 | -0.209 |
| Externalizing factor T3 | 0.094 | | 0.120 | 0.786 | 0.432 | -0.141 | 0.329 | 0.070 |
| ADHD factor T3 | 0.004 | | 0.092 | 0.043 | 0.966 | -0.176 | 0.184 | 0.005 |
| **ΔADHD at T2 ~ regressed on** |  | |  |  |  |  |  |  |
| ADHD factor T1 | -0.392 | | 0.047 | -8.294 | 0.000 | -0.485 | -0.299 | -0.422 |
| Internalizing factor T1 | 0.128 | | 0.038 | 3.380 | 0.001 | 0.054 | 0.203 | 0.146 |
| Externalizing factor T1 | -0.095 | | 0.049 | -1.956 | 0.051 | -0.190 | 0.000 | -0.089 |
| Prosociality factor T1 | 0.002 | | 0.032 | 0.071 | 0.943 | -0.060 | 0.065 | 0.003 |
| **ΔADHD at T3 ~ regressed on** |  | |  |  |  |  |  |  |
| ADHD factor T2 | -0.204 | | 0.087 | -2.336 | 0.019 | -0.375 | -0.033 | -0.233 |
| Internalizing factor T2 | 0.033 | | 0.077 | 0.430 | 0.667 | -0.117 | 0.183 | 0.041 |
| Externalizing factor T2 | 0.119 | | 0.096 | 1.240 | 0.215 | -0.069 | 0.306 | 0.102 |
| Prosociality factor T1 | 0.026 | | 0.072 | 0.366 | 0.715 | -0.114 | 0.167 | 0.031 |
| **ΔADHD at T4 ~ regressed on** |  | |  |  |  |  |  |  |
| ADHD factor T3 | -0.281 | | 0.104 | -2.696 | 0.007 | -0.485 | -0.077 | -0.340 |
| Internalizing factor T3 | 0.156 | | 0.099 | 1.578 | 0.114 | -0.038 | 0.350 | 0.206 |
| Externalizing factor T3 | -0.061 | | 0.128 | -0.477 | 0.634 | -0.313 | 0.190 | -0.046 |
| Prosociality factor T3 | -0.001 | | 0.100 | -0.006 | 0.995 | -0.196 | 0.195 | -0.001 |
| *Note: Δ indicates a change score, for instance ΔADHD at T2 indicates the latent variable that captures the change between the ADHD factor at T1 and the ADHD factor at T2.  **Note: *β* refers to the standardized regression coefficient. | | | | | | | | |
