# Supplementary material for: Common Cause Versus Dynamic Mutualism: An Empirical Comparison of Two Theories of Psychopathology in Two Large Longitudinal Cohorts
Source: Clin Psychol Sci. 2023 May 25;12(3):380–402. doi: 10.1177/21677026231162814 (PMC11136614; doi:10.1177/21677026231162814)
Supplement: sj-docx-20-cpx-10.1177_21677026231162814 – Supplemental material for Common Cause Versus Dynamic Mutualism: An Empirical Comparison of Two Theories of Psychopathology in Two Large Longitudinal Cohorts [file sj-docx-20-cpx-10.1177_21677026231162814.docx]

| Table S20  *Model comparison fit statistics for SHARE models with gender as a covariate* | | | | | |  |  |
| --- | --- | --- | --- | --- | --- | --- | --- |
| Model | χ2 | Df | RMSEA | CFI | SRMR | AIC | BIC |
| Common cause | 784.164 | 56 | 0.049 [0.046, 0.052] | 0.951 | 0.042 | 231185.823 | 231416.028 |
| Mutualism | 581.141 | 17 | 0.072 [00.67, 0.077] | 0.962 | 0.038 | 230905.137 | 231399.401 |
