# Supplementary material for: Common Cause Versus Dynamic Mutualism: An Empirical Comparison of Two Theories of Psychopathology in Two Large Longitudinal Cohorts
Source: Clin Psychol Sci. 2023 May 25;12(3):380–402. doi: 10.1177/21677026231162814 (PMC11136614; doi:10.1177/21677026231162814)
Supplement: sj-docx-21-cpx-10.1177_21677026231162814 – Supplemental material for Common Cause Versus Dynamic Mutualism: An Empirical Comparison of Two Theories of Psychopathology in Two Large Longitudinal Cohorts [file sj-docx-21-cpx-10.1177_21677026231162814.docx]

| Table S21A  *Self-feedback parameters for common cause model with gender as covariate (SHARE)* | | | | | | | |
| --- | --- | --- | --- | --- | --- | --- | --- |
| Regressions | Estimate | Std.Err | z-value | P(>\|z\|) | ci.lower | ci.upper | *β* |
| Δdepression at T2 regressed on ~ |  |  |  |  |  |  |  |
| Depression T1 | -0.202 | 0.030 | -6.672 | 0.000 | -0.261 | -0.142 | -0.399 |
| Δdepression at T3 regressed on ~ |  |  |  |  |  |  |  |
| Depression T2 | -0.117 | 0.028 | -4.198 | 0.000 | -0.172 | -0.063 | -0.190 |
| Δdepression at T4 regressed on ~ |  |  |  |  |  |  |  |
| Depression T3 | -0.077 | 0.023 | -3.290 | 0.001 | -0.122 | -0.031 | -0.143 |
| Δdepression at T5 regressed on ~ |  |  |  |  |  |  |  |
| Depression T4 | -0.133 | 0.023 | -5.704 | 0.000 | -0.179 | -0.088 | -0.232 |

| Table S21B  *Gender as covariate of depression factor at T1 (SHARE)* | | | | | | | |
| --- | --- | --- | --- | --- | --- | --- | --- |
| Regressions | Estimate | Std. Error | z-value | P(>\|z\|) | CI lower | CI upper | Beta |
| Depression T1 ~ Gender | 0.947 | 0.047 | 20.215 | 0.000 | 0.855 | 1.039 | 0.360 |
|  |  |  |  |  |  |  |  |
