# Supplementary material for: Common Cause Versus Dynamic Mutualism: An Empirical Comparison of Two Theories of Psychopathology in Two Large Longitudinal Cohorts
Source: Clin Psychol Sci. 2023 May 25;12(3):380–402. doi: 10.1177/21677026231162814 (PMC11136614; doi:10.1177/21677026231162814)
Supplement: sj-docx-22-cpx-10.1177_21677026231162814 – Supplemental material for Common Cause Versus Dynamic Mutualism: An Empirical Comparison of Two Theories of Psychopathology in Two Large Longitudinal Cohorts [file sj-docx-22-cpx-10.1177_21677026231162814.docx]

| Table S22  *Self-feedback and coupling parameters for dynamic mutualism model with gender as covariate at T1* (SHARE) | | | | | | | | |
| --- | --- | --- | --- | --- | --- | --- | --- | --- |
| Regression | Estimate | SE | z-value | p-value | CI_lower_ | CI_upper_ | Std.lv | β |
| Δaffect at T2 regressed on ~ | | | | | | | | |
| Affect T1 | -0.504 | 0.013 | -39.566 | 0.000 | -0.529 | -0.479 | -0.311 | -0.517 |
| Motivation T1 | 0.139 | 0.031 | 4.520 | 0.000 | 0.079 | 0.199 | 0.086 | 0.063 |
| Δaffect at T3 regressed on ~ | | | | | | | | |
| Affect T2 | -0.099 | 0.038 | -2.604 | 0.009 | -0.173 | -0.024 | -0.059 | -0.097 |
| Motivation T2 | 0.036 | 0.137 | 0.266 | 0.790 | -0.231 | 0.304 | 0.022 | 0.015 |
| Δaffect at T4 regressed on ~ | | | | | | | | |
| Affect T3 | -0.104 | 0.043 | -2.412 | 0.016 | -0.189 | -0.020 | -0.066 | -0.110 |
| Motivation T3 | 0.189 | 0.141 | 1.345 | 0.179 | -0.087 | 0.466 | 0.119 | 0.090 |
| Δaffect at T5 regressed on ~ | | | | | | | | |
| Affect T4 | 0.013 | 0.052 | 0.258 | 0.796 | -0.089 | 0.116 | 0.009 | 0.015 |
| Motivation T4 | -0.266 | 0.158 | -1.682 | 0.092 | -0.577 | 0.044 | -0.169 | -0.131 |
| Δmotivation at T2 regressed on ~ | | | | | | | | |
| Motivation T1 | -0.727 | 0.017 | -41.553 | 0.000 | -0.761 | -0.693 | -0.869 | -0.642 |
| Affect T1 | 0.057 | 0.006 | 9.049 | 0.000 | 0.045 | 0.069 | 0.068 | 0.113 |
| Δmotivation at T3 regressed on ~ | | | | | | | | |
| Motivation T2 | -0.108 | 0.084 | -1.276 | 0.202 | -0.273 | 0.058 | -0.123 | -0.087 |
| Affect T2 | 0.027 | 0.021 | 1.296 | 0.195 | -0.014 | 0.068 | 0.031 | 0.051 |
| Δmotivation at T4 regressed on ~ | | | | | | | | |
| Motivation T3 | 0.014 | 0.095 | 0.152 | 0.879 | -0.172 | 0.201 | 0.017 | 0.012 |
| Affect T3 | 0.001 | 0.026 | 0.054 | 0.957 | -0.049 | 0.052 | 0.002 | 0.003 |
| Δmotivation at T5 regressed on ~ | | | | | | | | |
| Motivation T4 | 0.070 | 0.101 | 0.690 | 0.490 | -0.129 | 0.269 | 0.083 | 0.064 |
| Affect T4 | -0.042 | 0.030 | -1.434 | 0.152 | -0.100 | 0.016 | -0.050 | -0.086 |

| Table S22B  *Gender as covariate of parcel scores at T1 (SHARE)* | | | | | | | |
| --- | --- | --- | --- | --- | --- | --- | --- |
| Regressions | Estimate | Std. Error | z-value | P(>\|z\|) | CI lower | CI upper | β |
| Affect T1 ~ Gender | 0.909 | 0.039 | 23.249 | 0.000 | 0.833 | 0.986 | 0.270 |
|  |  |  |  |  |  |  |  |
| Motivation T1 ~ Gender | 0.101 | 0.018 | 5.522 | 0.000 | 0.065 | 0.138 | 0.068 |
