# Supplementary material for: Common Cause Versus Dynamic Mutualism: An Empirical Comparison of Two Theories of Psychopathology in Two Large Longitudinal Cohorts
Source: Clin Psychol Sci. 2023 May 25;12(3):380–402. doi: 10.1177/21677026231162814 (PMC11136614; doi:10.1177/21677026231162814)
Supplement: sj-docx-23-cpx-10.1177_21677026231162814 – Supplemental material for Common Cause Versus Dynamic Mutualism: An Empirical Comparison of Two Theories of Psychopathology in Two Large Longitudinal Cohorts [file sj-docx-23-cpx-10.1177_21677026231162814.docx]

**Methodological Details**

We examined the developmental *p* factor that arises from a higher-order factor model and not the *p* factor that arises from a bifactor model. Our rationale is twofold. First, several concerns have been voiced regarding the use of bifactor models. These include concerns about the theoretical interpretability of specific factors (Bonifay, Lane, & Reise, 2017) and a propensity to overfit data, which urges caution when interpreting model fit indices (Murray & Johnson, 2013; Morgan et al., 2015). Second, it is not possible to estimate a dynamic mutualism model that can be used for comparison with a bifactor model. In a bifactor model the *p* factor directly explains a large component of the shared variance between all symptoms. A competing dynamic mutualism model needs to explain this shared variance through the causal interrelations between all symptoms. This needs more regression parameters than are possible to estimate with the degrees of freedom we have available. In a higher-order factor model, the *p* factor explains the shared variance between specific factors. A competing dynamic mutualism model needs to explain this shared variance through the causal interrelations between specific factors (not symptoms). This is possible. Directly comparing a dynamic mutualism model that specifies causal interrelations between specific factors with a bifactor common cause model, may result in comparing the ability of two different mechanisms to explain two different phenomena. As the bifactor model would explain the correlations between symptoms via a causal *p* factor, while the dynamic mutualism model would explain the correlations between specific factors via the causal interrelations between them. Hence, we will directly compare the higher-order factor model with the (only possible) dynamic mutualism model to mitigate the possibility that the models explain different phenomena.
