# Supplementary material for: Common Cause Versus Dynamic Mutualism: An Empirical Comparison of Two Theories of Psychopathology in Two Large Longitudinal Cohorts
Source: Clin Psychol Sci. 2023 May 25;12(3):380–402. doi: 10.1177/21677026231162814 (PMC11136614; doi:10.1177/21677026231162814)
Supplement: sj-docx-24-cpx-10.1177_21677026231162814 – Supplemental material for Common Cause Versus Dynamic Mutualism: An Empirical Comparison of Two Theories of Psychopathology in Two Large Longitudinal Cohorts [file sj-docx-24-cpx-10.1177_21677026231162814.docx]

| 1. Internalizing items | 1. Prosociality items (reverse coded) |  |  |
| --- | --- | --- | --- |
| 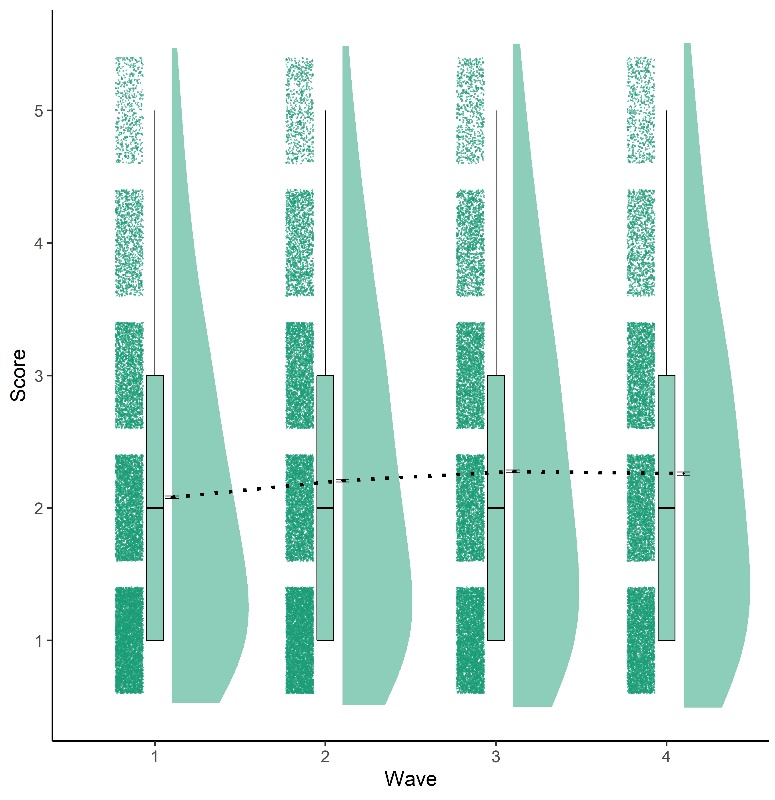 | 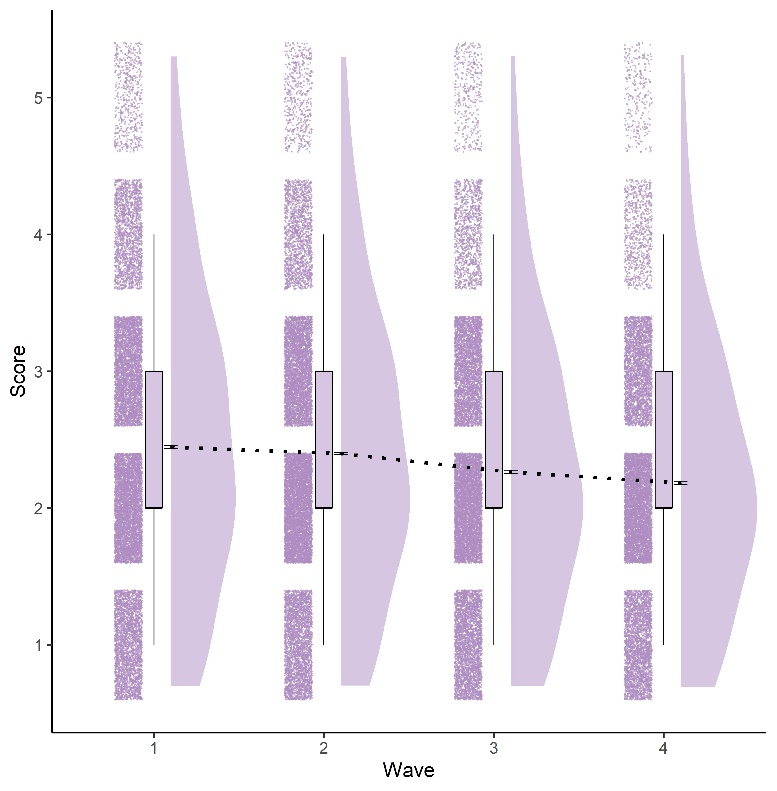 | |  |
| 1. ADHD items | 1. Externalizing items |  |  |
| 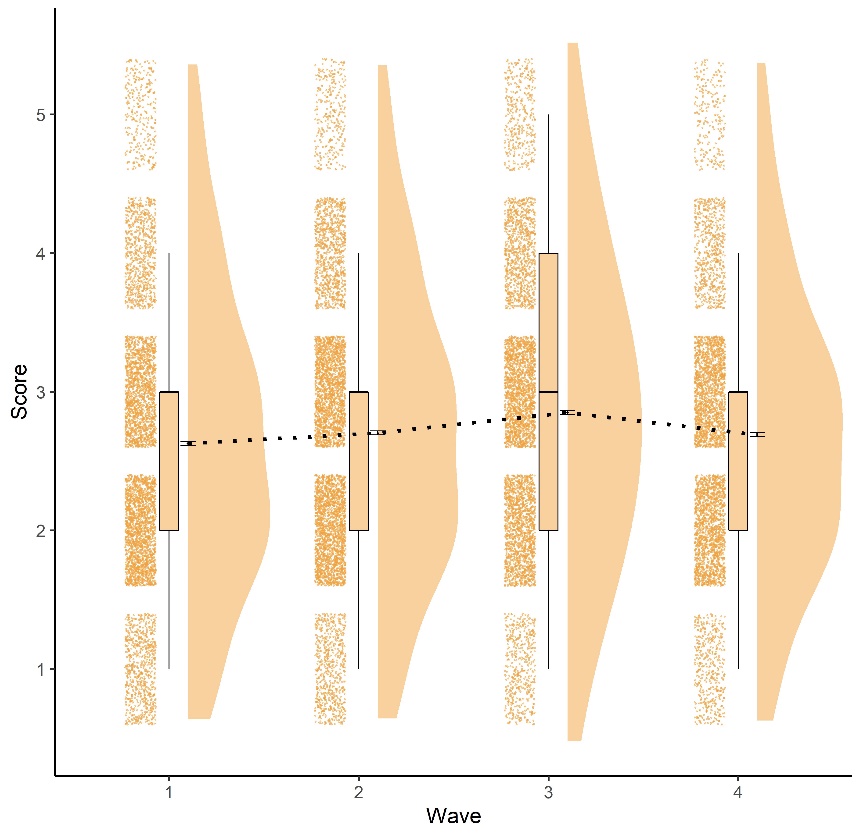 | 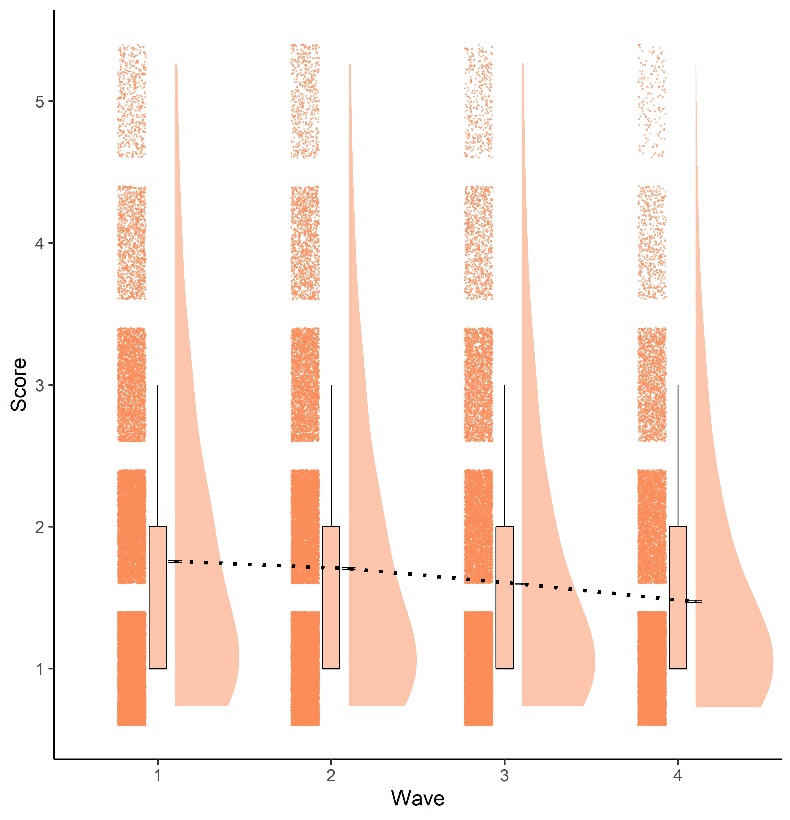 | | |
| *Figure S1*. Raincloud plots of items grouped by factor structure of zproso dataset. Items are rated on a 5-point Likert scale. Higher scores on all domains indicate a higher degree of psychopathology. The black horizontal bars at the base (approximately middle) of each density plot represent the standard error of the mean. The dashed black lines passing through subsequent waves indicate changes in mean severity of psychopathology over time. | |  |  |
