# Supplementary material for: Common Cause Versus Dynamic Mutualism: An Empirical Comparison of Two Theories of Psychopathology in Two Large Longitudinal Cohorts
Source: Clin Psychol Sci. 2023 May 25;12(3):380–402. doi: 10.1177/21677026231162814 (PMC11136614; doi:10.1177/21677026231162814)
Supplement: sj-docx-25-cpx-10.1177_21677026231162814 – Supplemental material for Common Cause Versus Dynamic Mutualism: An Empirical Comparison of Two Theories of Psychopathology in Two Large Longitudinal Cohorts [file sj-docx-25-cpx-10.1177_21677026231162814.docx]

**
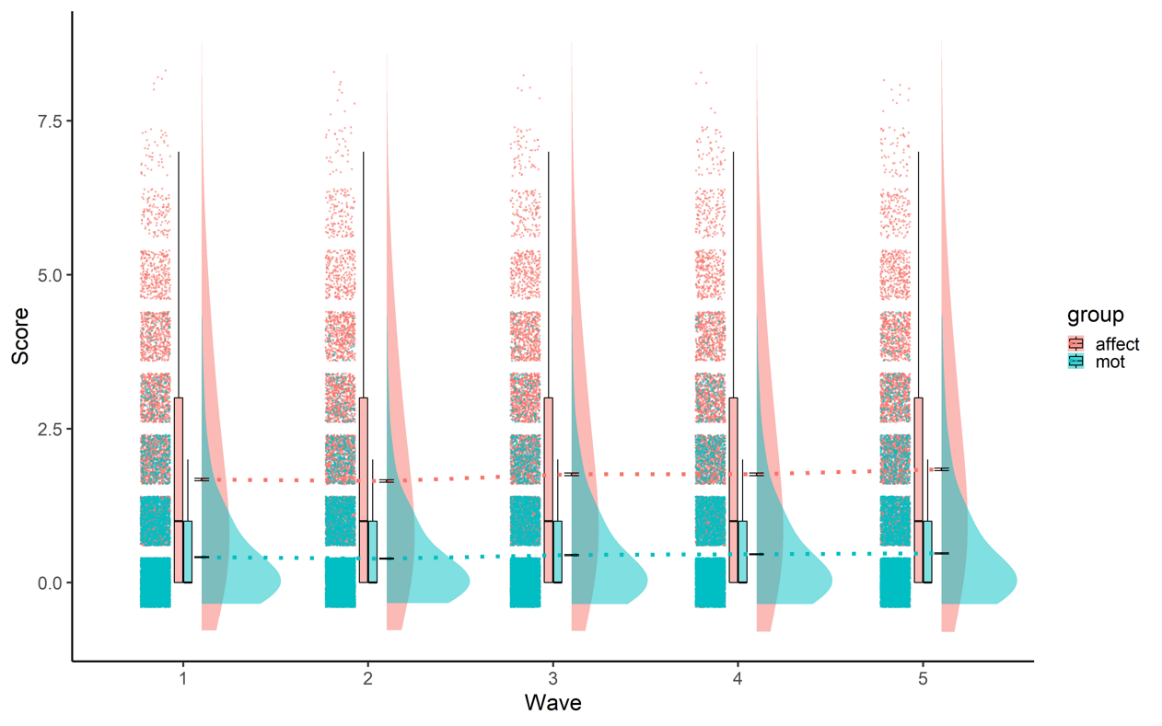
**

*Figure S2.* Raincloud plot of depression items within the two parcels extracted from the SHARE data. The first parcel representing the “affective suffering” construct, included the items of sadness, suicidality, guilt, sleeplessness, irritability, appetite, fatigue, and tearfulness (maximum score 8). The second parcel representing the “motivation” construct, included the items of pessimism, interest, concentration, and enjoyment (maximum score 4). Density plots represent the prevalence of affective suffering and motivation items at each time point, with a higher score indicating higher prevalence of items in the sample. The black horizontal lines at the base of the density plots (approximately at the midpoint) represent the standard error of the mean. The dashed colored lines passing through subsequent waves display changes in the mean prevalence of symptoms over time.
