# Supplementary material for: Common Cause Versus Dynamic Mutualism: An Empirical Comparison of Two Theories of Psychopathology in Two Large Longitudinal Cohorts
Source: Clin Psychol Sci. 2023 May 25;12(3):380–402. doi: 10.1177/21677026231162814 (PMC11136614; doi:10.1177/21677026231162814)
Supplement: sj-docx-3-cpx-10.1177_21677026231162814 – Supplemental material for Common Cause Versus Dynamic Mutualism: An Empirical Comparison of Two Theories of Psychopathology in Two Large Longitudinal Cohorts [file sj-docx-3-cpx-10.1177_21677026231162814.docx]

| Table S3  *Change score variances for dynamic mutualism model (z-proso)* | | | | | | | |
| --- | --- | --- | --- | --- | --- | --- | --- |
| Change scores | Estimate | Std.Err | z-value | P(>\|z\|) | ci.lower | ci.upper | *β* |
| Δinternalizing at T2 | 0.416 | 0.030 | 13.805 | 0.000 | 0.357 | 0.475 | 0.839 |
| Δinternalizing at T3 | 0.415 | 0.033 | 12.561 | 0.000 | 0.350 | 0.480 | 0.891 |
| Δinternalizing at T4 | 0.464 | 0.040 | 11.461 | 0.000 | 0.385 | 0.543 | 0.880 |
| Δexternalizing at T2 | 0.192 | 0.017 | 11.226 | 0.000 | 0.158 | 0.225 | 0.714 |
| Δexternalizing at T3 | 0.145 | 0.018 | 8.093 | 0.000 | 0.110 | 0.180 | 0.744 |
| Δexternalizing at T4 | 0.086 | 0.012 | 7.160 | 0.000 | 0.062 | 0.109 | 0.610 |
| Δprosociality at T2 | 0.360 | 0.023 | 15.392 | 0.000 | 0.314 | 0.406 | 0.742 |
| Δprosociality at T3 | 0.344 | 0.025 | 13.496 | 0.000 | 0.294 | 0.394 | 0.848 |
| Δprosociality at T4 | 0.272 | 0.021 | 12.826 | 0.000 | 0.231 | 0.314 | 0.660 |
| ΔADHD at T2 | 0.337 | 0.026 | 13.039 | 0.000 | 0.286 | 0.388 | 0.818 |
| ΔADHD at T3 | 0.366 | 0.031 | 11.710 | 0.000 | 0.305 | 0.428 | 0.886 |
| ΔADHD at T4 | 0.311 | 0.032 | 9.829 | 0.000 | 0.249 | 0.373 | 0.787 |

*Note: Δ represents the latent variable that captures change between time points, e.g. ΔADHD at T2 represents the change between the ADHD factor scores at T1 and the ADHD factor scores at T2.
