# Supplementary material for: Common Cause Versus Dynamic Mutualism: An Empirical Comparison of Two Theories of Psychopathology in Two Large Longitudinal Cohorts
Source: Clin Psychol Sci. 2023 May 25;12(3):380–402. doi: 10.1177/21677026231162814 (PMC11136614; doi:10.1177/21677026231162814)
Supplement: sj-docx-4-cpx-10.1177_21677026231162814 – Supplemental material for Common Cause Versus Dynamic Mutualism: An Empirical Comparison of Two Theories of Psychopathology in Two Large Longitudinal Cohorts [file sj-docx-4-cpx-10.1177_21677026231162814.docx]

| Table S4  *Self-feedback parameters for common cause model (SHARE)* | | | | | | | |
| --- | --- | --- | --- | --- | --- | --- | --- |
| Regressions | Estimate | Std.Err | z-value | P(>\|z\|) | ci.lower | ci.upper | *β* |
| Δdepression at T2 regressed on ~ |  |  |  |  |  |  |  |
| Depression T1 | -0.271 | 0.032 | -8.537 | 0.000 | -0.333 | -0.209 | -0.455 |
| Δdepression at T3 regressed on ~ |  |  |  |  |  |  |  |
| Depression T2 | -0.179 | 0.040 | -4.446 | 0.000 | -0.257 | -0.100 | -0.244 |
| Δdepression at T4 regressed on ~ |  |  |  |  |  |  |  |
| Depression T3 | -0.090 | 0.033 | -2.475 | 0.006 | -0.154 | -0.026 | -0.142 |
| Δdepression at T5 regressed on ~ |  |  |  |  |  |  |  |
| Depression T4 | -0.167 | 0.026 | -6.463 | 0.000 | -0.217 | -0.116 | -0.296 |

*Note: Δ represents the latent variable that captures change between time points, e.g. Δdepression at T2 represents the change between the Depression factor scores at T1 and the Depression factor scores at T2.
