# Supplementary material for: Common Cause Versus Dynamic Mutualism: An Empirical Comparison of Two Theories of Psychopathology in Two Large Longitudinal Cohorts
Source: Clin Psychol Sci. 2023 May 25;12(3):380–402. doi: 10.1177/21677026231162814 (PMC11136614; doi:10.1177/21677026231162814)
Supplement: sj-docx-5-cpx-10.1177_21677026231162814 – Supplemental material for Common Cause Versus Dynamic Mutualism: An Empirical Comparison of Two Theories of Psychopathology in Two Large Longitudinal Cohorts [file sj-docx-5-cpx-10.1177_21677026231162814.docx]

| Table S5  *Change score variances for common cause model and dynamic mutualism model* | | | | | | | |
| --- | --- | --- | --- | --- | --- | --- | --- |
| Common cause model | | | | | | | |
| Change score | Estimate | Std.Err | z-value | P(>\|z\|) | ci.lower | ci.upper | *β* |
| Δpfactor T2 | 0.300 | 0.050 | 6.063 | 0.000 | 0.203 | 0.398 | 0.793 |
| Δpfactor T3 | 0.438 | 0.048 | 9.180 | 0.000 | 0.345 | 0.532 | 0.941 |
| Δpfactor T4 | 0.399 | 0.040 | 9.876 | 0.000 | 0.320 | 0.478 | 0.980 |
| Δpfactor T5 | 0.360 | 0.051 | 7.022 | 0.000 | 0.260 | 0.461 | 0.912 |
| Dynamic mutualism model | | | | | | | |
| Change scores | Estimate | Std. Err | z-value | P(>\|z\|) | ci.lower | ci.upper | *β* |
| Δaffect T2 | 1.978 | 0.042 | 46.649 | 0.000 | 1.894 | 2.061 | 0.752 |
| Δaffect T3 | 2.592 | 0.083 | 31.100 | 0.000 | 2.429 | 2.756 | 0.917 |
| Δaffect T4 | 2.342 | 0.076 | 30.842 | 0.000 | 2.193 | 2.491 | 0.927 |
| Δaffect T5 | 2.473 | 0.135 | 18.383 | 0.000 | 2.210 | 2.737 | 0.999 |
| Δmotivation T2 | 0.439 | 0.015 | 30.211 | 0.000 | 0.411 | 0.468 | 0.628 |
| Δmotivation T3 | 0.701 | 0.051 | 13.732 | 0.000 | 0.601 | 0.801 | 0.921 |
| Δmotivation T4 | 0.770 | 0.070 | 10.938 | 0.000 | 0.632 | 0.908 | 1.015 |
| Δmotivation T5 | 0.747 | 0.072 | 10.412 | 0.000 | 0.606 | 0.888 | 1.055 |

*Note: Δ represents the latent variable that captures change between time points, e.g. Δpfactor T2 represents the change between the p-factor scores at T1 and the p-factor scores at T2.
