# Supplementary material for: Common Cause Versus Dynamic Mutualism: An Empirical Comparison of Two Theories of Psychopathology in Two Large Longitudinal Cohorts
Source: Clin Psychol Sci. 2023 May 25;12(3):380–402. doi: 10.1177/21677026231162814 (PMC11136614; doi:10.1177/21677026231162814)
Supplement: sj-docx-6-cpx-10.1177_21677026231162814 – Supplemental material for Common Cause Versus Dynamic Mutualism: An Empirical Comparison of Two Theories of Psychopathology in Two Large Longitudinal Cohorts [file sj-docx-6-cpx-10.1177_21677026231162814.docx]

| Table S6  *Self-feedback and coupling parameters for dynamic mutualism model* (SHARE) | | | | | | | | |
| --- | --- | --- | --- | --- | --- | --- | --- | --- |
| Regression | Estimate | SE | z-value | p-value | CI_lower_ | CI_upper_ | Std.lv | Std.all |
| Δaffect at T2 regressed on ~ | | | | | | | | |
| Affect T1 | -0.504 | 0.013 | -39.559 | 0.000 | -0.529 | -0.479 | -0.311 | -0.517 |
| Motivation T1 | 0.140 | 0.031 | 4.539 | 0.000 | 0.079 | 0.200 | 0.086 | 0.064 |
| Δaffect at T3 regressed on ~ | | | | | | | | |
| Affect T2 | -0.099 | 0.038 | -2.611 | 0.009 | -0.173 | -0.025 | -0.059 | -0.097 |
| Motivation T2 | 0.038 | 0.137 | 0.278 | 0.781 | -0.230 | 0.306 | 0.023 | 0.016 |
| Δaffect at T4 regressed on ~ | | | | | | | | |
| Affect T3 | -0.106 | 0.043 | -2.439 | 0.015 | -0.190 | -0.021 | -0.066 | -0.112 |
| Motivation T3 | 0.191 | 0.141 | 1.356 | 0.175 | -0.085 | 0.467 | 0.120 | 0.091 |
| Δaffect at T5 regressed on ~ | | | | | | | | |
| Affect T4 | 0.015 | 0.052 | 0.281 | 0.778 | -0.088 | 0.117 | 0.009 | 0.016 |
| Motivation T4 | -0.268 | 0.159 | -1.693 | 0.091 | -0.579 | 0.042 | -0.171 | -0.132 |
| Δmotivation at T2 regressed on ~ | | | | | | | | |
| Motivation T1 | -0.727 | 0.018 | -41.540 | 0.000 | -0.761 | -0.693 | -0.869 | -0.642 |
| Affect T1 | 0.057 | 0.006 | 9.033 | 0.000 | 0.045 | 0.069 | 0.068 | 0.113 |
| Δmotivation at T3 regressed on ~ | | | | | | | | |
| Motivation T2 | -0.107 | 0.084 | -1.267 | 0.205 | -0.273 | 0.059 | -0.123 | -0.087 |
| Affect T2 | 0.027 | 0.021 | 1.300 | 0.194 | -0.194 | 0.068 | 0.031 | 0.052 |
| Δmotivation at T4 regressed on ~ | | | | | | | | |
| Motivation T3 | 0.051 | 0.095 | 0.162 | 0.871 | -0.171 | 0.202 | 0.018 | 0.013 |
| Affect T3 | 0.001 | 0.026 | 0.029 | 0.977 | -0.050 | 0.051 | 0.001 | 0.001 |
| Δmotivation at T5 regressed on ~ | | | | | | | | |
| Motivation T4 | 0.068 | 0.101 | 0.670 | 0.503 | -0.131 | 0.266 | 0.081 | 0.062 |
| Affect T4 | -0.041 | 0.030 | -1.401 | 0.161 | -0.099 | 0.017 | -0.049 | -0.084 |

*Note: Δ represents the latent variable that captures change between time points, e.g. Δaffect at T2 represents the change between the affect-parcel scores at T1 and the affect-parcel scores at T2.
