# Supplementary material for: Common Cause Versus Dynamic Mutualism: An Empirical Comparison of Two Theories of Psychopathology in Two Large Longitudinal Cohorts
Source: Clin Psychol Sci. 2023 May 25;12(3):380–402. doi: 10.1177/21677026231162814 (PMC11136614; doi:10.1177/21677026231162814)
Supplement: sj-docx-7-cpx-10.1177_21677026231162814 – Supplemental material for Common Cause Versus Dynamic Mutualism: An Empirical Comparison of Two Theories of Psychopathology in Two Large Longitudinal Cohorts [file sj-docx-7-cpx-10.1177_21677026231162814.docx]

| Table S7  *Model comparison fit statistics SHARE data* | | | | | |
| --- | --- | --- | --- | --- | --- |
| Exploratory models | | | | | |
| Model | χ2 | df | RMSEA | CFI | SRMR |
| Common cause^1^ | 394.814 | 40 | 0.040 [0.037, 0.043] | 0.977 | 0.030 |
| Common cause^2^ | 233.838 | 36 | 0.032 [0.028, 0.036] | 0.987 | 0.020 |
| Dynamic mutualism^3^ | 42.735 | 6 | 0.034 [ 0.025, 0.044] | 0.998 | 0.010 |
| Note: 1 = Common model with residual change score covariance over time. 2= Common cause model with residual change score covariance over time and direct age effects on change. 3 = Dynamic mutualism model with age directly influencing change scores and coupling parameters constrained to equality to over-identify model. | | | | | |
