# Supplementary material for: Common Cause Versus Dynamic Mutualism: An Empirical Comparison of Two Theories of Psychopathology in Two Large Longitudinal Cohorts
Source: Clin Psychol Sci. 2023 May 25;12(3):380–402. doi: 10.1177/21677026231162814 (PMC11136614; doi:10.1177/21677026231162814)
Supplement: sj-docx-8-cpx-10.1177_21677026231162814 – Supplemental material for Common Cause Versus Dynamic Mutualism: An Empirical Comparison of Two Theories of Psychopathology in Two Large Longitudinal Cohorts [file sj-docx-8-cpx-10.1177_21677026231162814.docx]

| Table S8  *Deviations from preregistration* | | | |
| --- | --- | --- | --- |
| Dataset | Planned to | Deviation | Rationale |
| SHARE | Use four symptom items as indicators. | Used item parcels as indicators. | To normalize data for maximum likelihood estimation. |
|  | Use data from all persons in sample. | Used only the persons that had at least 1 measurement on the EURO-D scale across all waves. | We wanted to assess developmental changes over time and most cases only had data on one wave. This would have resulted in more than 70 percent missing data. Hence, only analyzing a subset of this data was deemed most defensible. |
| z-proso | We reported that the sample size for the 4 waves used was 1532. | The actual sample size was 1482. | — |
|  | Use a four-factor EFA to identify item content of factors. | We specified the item content of the four factors based on theory. | The four-factor EFA showed a divergent optimal structure throughout the four waves. To specify a homogeneous factor model that is most generalizable, we relied on the conceptual congruence of the items instead. |
